# Genetic Ablation and Multi‐Omics Profiling Reveal CEP55 as a Key Driver of Tumorigenesis in Diverse Cancer Models

**DOI:** 10.1002/advs.76782

**Published:** 2026-08-10

**Authors:** Behnam Rashidieh, Yuanhao Yang, Amanda Louise Bain, Phillip Theron, Catharina Vosloo, Sahar Keshvari, Parinaz Ahangar, Brydie Bowden, Simon M. Tria, Ben B. Wang, Sandra Isenmann, Nicholas J. Westra van Holthe, Zherui Xiong, Hani Vu, Sowmya Sharma, Asmerom Sengal, J. Alejandro Lopez, John Finnie, Antonella Papa, Pascal H.G. Duijf, Quan Nguyen, Pirjo M. Apaja, Kum Kum Khanna

**Affiliations:** ^1^ Mater Research Institute The University of Queensland Translational Research Institute Brisbane QLD Australia; ^2^ Cancer Research Program QIMR Berghofer Medical Research Institute Brisbane QLD Australia; ^3^ Australian Institute for Bioengineering and Nanotechnology The University of Queensland Brisbane Queensland Australia; ^4^ School of Environment and Science Griffith University Brisbane QLD Australia; ^5^ Department of Molecular & Cellular Biology University of Adelaide School of Biological Sciences Adelaide SA Australia; ^6^ South Australian Health and Medical Research Institute Adelaide SA Australia; ^7^ ARC Centre of Excellence in Quantum Biotechnology St Lucia Queensland Australia; ^8^ School of Biomedicine Faculty of Health and Medical Sciences University of Adelaide Adelaide SA Australia; ^9^ South Australian immunogenomics Cancer Institute The University of Adelaide Adelaide South Australia Australia; ^10^ Monash Biomedicine Discovery Institute and Department of Biochemistry and Molecular Biology Monash University Melbourne VIC Australia; ^11^ Centre for Cancer Biology Clinical and Health Sciences University of South Australia & SA Pathology Adelaide SA Australia; ^12^ Flinders University College of Public Health and Medicine Bedford Park SA Australia

**Keywords:** CEP55, integrin and PI3K/AKT pathways, PTEN, tumorigenesis

## Abstract

CEP55, a centrosomal protein frequently overexpressed in multiple cancers, is implicated in regulation of genomic instability and PI3K/AKT pathway activation, yet its role in tumor progression and the tumor microenvironment remains incompletely defined. Here, we developed an inducible Cep55 knockout (KO) mouse model and complementary primary, immortalized, and transformed mouse embryonic fibroblast (MEF) systems and employ integrative multi‐omics analyses to define CEP55‐driven mechanisms and validated findings in human cancer datasets. Cep55 deletion is well tolerated in adult tissues but significantly delays tumor onset and progression in E1A/Ras‐driven models and prolongs survival in PTEN‐deficient cancer models. Mechanistically, spatial transcriptomic and proteomic analyses revealed that CEP55 regulates extracellular matrix (ECM) remodeling, integrin signaling, and cell adhesion pathways, AKT/ERK signaling and cellular stress responses. Importantly, loss of Cep55 also reprograms the tumor immune microenvironment, reducing tumor‐supportive macrophage polarization and enhancing antigen‐specific CD8^+^ T‐cell responses in vitro. Collectively, these findings demonstrate that CEP55 is dispensable for adult tissue homeostasis but plays a critical role in tumor progression by coordinating oncogenic signaling, extracellular matrix dynamics, and tumor–immune interactions and highlight it as a potential therapeutic target in aggressive and immune‐resistant cancers.

## Graphical abstract

Genetic ablation of Cep55 in Pten‐deficient mouse models delays tumorigenesis. Integrated multi‐omics analyses (proteomics, phosphoproteomics, and spatial transcriptomics) reveal that CEP55 regulates oncogenic signaling (RAS/ERK, PI3K/AKT), integrin/FAK‐mediated adhesion, extracellular matrix (ECM) remodeling, and endocytosis. CEP55 loss also reshapes the tumor microenvironment by modulating immune responses. Cross‐species integration with human cancer datasets (TCGA) demonstrates conserved co‐regulation of immune, integrin/adhesion, and endosomal programs, highlighting CEP55 as a central driver of tumor progression and tumor–immune interactions.

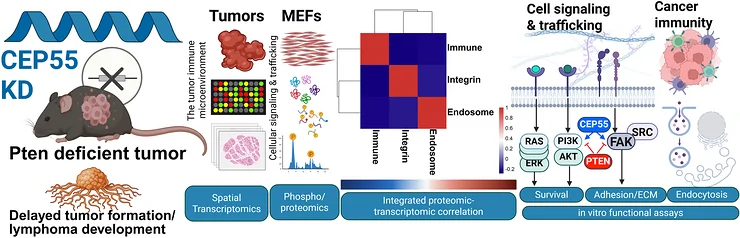

**Abbreviations**
AT3: Mouse mammary carcinoma cell line
CEP55: Centrosomal protein of 55 kDa
cKO: Conditional KO (inducible)
CP DKD: Cep55‐Pten double knockdown
E1A/Ras: Adenovirus E1A + Rat sarcoma virus
ECM: Extracellular matrix
EV: Empty vector
GEMM: Genetically Engineered Mouse Models
GFOGER: Represents the six‐letter amino acid sequence
Het: Heterozygous
Hom: Homozygous
IMM: immortalized
KD: Knock‐down
KO: Knockout (constitutive)
MEFs: Mouse Embryonic Fibroblasts
OE: Overexpression
OVA: Ovalbumin
PIC: Polyisocyanopeptide
pMEFs: Primary MEFs
Pten KD: Pten Knockdown
ROI: Region of interest
SV40: Simian virus 40 Large T Antigen
TMA: Tissue microarray
TME: Tumor microenvironment
WB: Western blot
Wt: Wild‐type

## 1 Background

Centrosomal protein 55 (*CEP55*) is a key regulator of cytokinesis. During the final stage of cell division, CEP55 recruits Endosomal Sorting Complex Required for Transport (ESCRT) components, including tumor‐susceptibility gene 101 (TSG101) and ALG‐2‐interacting protein X (ALIX; PDCD6IP), to the midbody to mediate daughter cell abscission [1, 2]. ALIX and ESCRT‐I/II cooperate to recruit the ESCRT‐III subunit CHMP4B, facilitating abscission [3]. Despite these interactions, CEP55 is not a core component of the canonical endosomal ESCRT machinery and instead acts as a context‐specific recruiter during cytokinetic abscission [4].

Beyond its canonical role in cytokinesis, emerging evidence indicates that CEP55 contributes to membrane trafficking and extracellular vesicle dynamics. CEP55 can be incorporated into and transferred via exosomes, where it is recruited to CD63‐positive secretory endosomes, implicating it in intercellular communication and microenvironmental signaling [5]. Consistent with this, CEP55 has been linked to immune infiltration and regulation of tumor–immune interactions across multiple cancer types, suggesting broader roles in shaping the tumor microenvironment beyond cell‐intrinsic proliferation pathways [6, 7].

Given the fundamental role of *CEP55* in the regulation of cell proliferation, it is not surprising that its dysregulation has been implicated in the development and progression of numerous human cancers. CEP55 is overexpressed across a wide range of malignancies, including breast, lung, colon, liver cancers, T‐cell lymphoma and is frequently associated with poor prognosis, advanced disease stage, metastasis and resistance to immunotherapy [8]. While largely silenced in adult tissues, CEP55 is aberrantly re‐expressed in many cancers and is classified as a cancer‐testis antigen. Functionally, it promotes oncogenic signaling by interacting with PIK3CA, leading to enhanced PI3K/AKT pathway activity and enhanced survival and proliferation [9, 10, 11, 12].

Overexpression of CEP55 alone is sufficient to induce tumorigenesis in a genetically engineered mouse model, promoting activation of PI3K/AKT pathway, mitotic defects and genomic instability [11]. Conversely, CEP55 knockdown or silencing impairs cancer cell proliferation and viability in cancer cell lines. These findings position CEP55 as an oncogenic driver and an attractive candidate for targeted cancer therapy.

In contrast, PTEN (the phosphatase and tensin homolog) is one of the most frequently inactivated tumor suppressors in human cancer [13]. PTEN antagonizes the PI3K/AKT pathway by dephosphorylating PIP3 to PIP2, thereby inhibiting AKT activation and downstream proliferative signaling. Loss of PTEN function via mutations, genomic deletion, or epigenetic silencing results in constitutive activation of PI3K/AKT signaling, promoting enhanced cancer cell survival, proliferation, angiogenesis and invasiveness. Biallelic PTEN Loss (deletions or mutations) is observed in numerous malignancies, including aggressive subsets of breast cancer, prostate, glioblastoma, and endometrial carcinoma [14]. In mouse models, systemic heterozygous *Pten* deletion drives the rapid development of multifocal tumors (notably lymphomas and carcinomas), underscoring its broad tumor‐suppressive function [15, 16]. Clinically, PTEN‐deficient tumors are associated with poor outcomes and frequent resistance to PI3K/AKT/mTOR inhibitors [17, 18]. PTEN loss fosters an immunosuppressive tumor microenvironment, reducing responses to immune checkpoint blockade therapies [19]. These challenges underscore the urgent need for alternative therapeutic strategies in PTEN‐deficient cancers [20].

Notably, oncogenic activity of CEP55 and the effects of PTEN loss converge on the PI3K/AKT pathway. CEP55‐driven PI3K stabilization and AKT activation functionally mimic the hyperactivation of this pathway caused by PTEN loss. In addition, given its emerging links to membrane dynamics and extracellular signaling, CEP55 may further contribute to tumor progression by influencing cell adhesion, extracellular matrix interactions, and tumor–microenvironment crosstalk. We therefore hypothesized that disrupting CEP55 in a PTEN‐deficient context could attenuate tumor progression through both cell‐intrinsic signaling and microenvironmental mechanisms.

In this study, we developed a novel tamoxifen‐inducible Cep55 conditional knockout mouse and evaluated the impact of Cep55 loss on cellular transformation and tumorigenesis both in vitro and in vivo. We show that Cep55 loss exerts multifaceted effects by remodeling endosomal pathway proteins, including ESCRTs, and altering fatty acid metabolism, immune pathways, and adhesion proteins. We demonstrate that Cep55 loss disrupts integrin endocytosis, cell adhesion, migration, and downstream signaling competence, linking Cep55 deficiency to broad cellular changes that mitigate tumorigenic features. The study employs genetically engineered mouse models combining inducible *Cep55 deletion* with *Pten* loss. Specifically, we investigated whether dual Cep55‐Pten knockdown (CP‐DKD) would hamper tumorigenesis in mice with loss of Pten alone. Our findings provide crucial insights into CEP55's role in cancer development and suggest that targeting CEP55 may represent a viable strategy for *PTEN*‐deficient cancers.

## 2 Methodology

### 2.1 Mouse Models

A *Cep55* floxed allele was engineered (knockout‐first allele construct) and crossed with *Rosa26‐Cre^ERT2^* mice to generate an inducible knockout (Cep55^Fl/Fl^; Cre^ERT2+/–^). Tamoxifen (single intraperitoneal dose, 1 mg) was administered to 6–8‐week‐old mice to induce global Cep55 deletion (cKO). A *Pten* ^Fl/Fl^ line was crossed to Cep55 cKO mice to produce Cep55/Pten double conditional knockouts. Mice were monitored for health for up to 160 days post‐induction of tamoxifen. Stable Cep55 knockout was confirmed by PCR and immunoblot. All animal procedures were approved by institutional ethics committees, QIMR Berghofer Animal Ethics Committee (A0707‐606 M).

### 2.2 Tumor‐Prone Pten Loss Model

Cep55^Fl/Fl^; Pten^Fl/Fl^; Cre^ERT2+/–^ mice and Pten^Fl/Fl^; Cre^ERT2+/–^ only controls (6–8 weeks old) were induced with tamoxifen to delete both genes (Cep55‐Pten DKD) or Pten KD alone. Mice were monitored for morbidity. Moribund or ≥160 days post‐tamoxifen induction survivors (3) were necropsied. Tissues (thymus, lymph nodes, spleen, liver, etc.) were examined by a veterinary pathologist. Histopathological analysis, including Hematoxylin and Eosin (H&E) staining and immunohistochemistry (IHC), was performed as previously described, [21] on tumor tissues to assess tumor morphology and protein expression. Tumors were classified and incidence recorded. Kaplan–Meier survival curves were generated for each cohort.

### 2.3 Transplant Cancer Cell‐Derived Model

10^6^ E1A/Ras‐transformed MEFs (Cep55 Wt or KO) 1:1 in Matrigel were injected subcutaneously into NOD/SCID (Non‐Obese Diabetic/Severe Combined Immunodeficiency) mice (n = 6 per group). Mice were examined thrice weekly for palpable tumors and sacrificed at endpoint tumor size (∼1000 mm^3^). Tumor volume was measured by calipers. Kaplan–Meier analysis compared tumor‐free survival between groups. Harvested tumors were formalin‐fixed for histology.

### 2.4 Cell Culture and Transformation

Primary mouse embryonic fibroblasts (pMEFs) were isolated from E13.5 Cep55^+/+^ and Cep55^–/–^ embryos (the latter obtained from heterozygous intercrosses). MEFs were cultured in DMEM + 20% FBS and either used as primary cells or immortalized by SV40 large T antigen or transformed by adenoviral E1A and oncogenic Ras (Ras^V12^) to model stepwise tumorigenesis using lentiviral packaging system.

### 2.5 Single Vesicular pH Trafficking Microscopy

Integrin and other cargo tracking using pH‐sensitive fluorophores, as well as analysis, was performed as previously described [23]. ALIX minimal Cep55‐interacting peptide (sequence GPPSLNY) [4] and control (GPASLNA incorporating alanine substitution) were synthesized with N‐terminal myristylation and C‐terminal amidation to enhance membrane association and peptide stability. Cells were treated with 20 µM peptides in serum free medium before the experiment. Live‐cell image acquisition was carried out Nikon TI‐E inverted microscope, using triggered acquisition, and analyzed with NIS‐Elements (Nikon, Japan).

### 2.6 Cellular Functional Assays

Proliferation was measured in real‐time (IncuCyte live‐cell imaging) over 5–7 days as previously described [24]. Colony formation was assessed in 2D (plating 500 cells/well, 14 days) and 3D soft agar and Happy Cell suspension medium assays (14 days). Wound‐healing migration assays and Transwell invasion assays (Matrigel‐coated chambers) were performed to evaluate cellular plasticity. An adhesion assay measured cell attachment to collagen‐coated plates. A custom invasion assay was established using a polyisocyanopeptide (PIC)‐based hydrogel modified with the collagen mimetic peptide GFOGER (PIC‐GFOGER), (GFOGER: Glycine‐Phenylalanine‐Hydroxyproline‐Glycine‐Glutamic Acid‐Arginine) within ultra‐low attachment plates [25].

### 2.7 Proteomic and Phosphoproteomic

Quantitative mass spectrometry was performed on Wt vs. Cep55‐KO MEFs (primary, SV40‐immortalized, and E1A/Ras‐transformed) as previously described [21]. Total protein lysates were trypsin‐digested and analyzed by high‐resolution LC‐MS/MS. Proteomic and phosphoproteomic analyses were performed using a tandem mass tag (TMT)‐based quantitative mass‐spectrometry workflow adapted from established QIMR Berghofer proteomics facility protocols, with phosphopeptide enrichment based on the EasyPhos TiO_2_ strategy which defines enrichment specificity as the number of unique phosphopeptides identified divided by the total number of unique peptides identified in the enriched fraction [26]. (See the extended methods).

### 2.8 Spatial Transcriptomics

To investigate gene expression changes in splenic lymphomas, we performed 10× Genomics Visium spatial transcriptomics on FFPE spleen sections from Pten‐KD and Cep55‐Pten‐DKD mice (n = 3 tumors per group). FFPE tissue sections were processed following the manufacturer's instructions (Visium CytAssist Tissue Preparation Guide, CG000518). Briefly, 5 µm tissue sections mounted on glass slides were stained with hematoxylin and eosin (H&E) and imaged using the Aperio XT Brightfield Automated Slide Scanner (Leica, Germany) at 20× magnification. After imaging, tissue sections were destained and decrosslinked, then hybridized with mouse transcriptome probes using the 10× Genomics Mouse Transcriptome 6.5 mm kit (PN #1000521, #1000365).

Probe extension and library construction were performed according to the standard Visium CytAssist FFPE workflow (Visium Spatial Gene Expression User Guide, CG000495). The libraries were sequenced using an Illumina NextSeq 2000 (Illumina, USA) with paired‐end dual indexing: 28 cycles for Read 1, 10 cycles for i7, 10 cycles for i5, and 50 cycles for Read 2. Sequencing data were demultiplexed using bcl2fastq v2.20.0.422 (Illumina). Reads were aligned and mapped to the H&E images using Space Ranger v2.1.1 (10× Genomics) and the mm10 mouse reference genome (refdata‐gex‐mm10‐2020‐A). Following quality control to exclude low‐quality spots using the Seurat v5 pipeline, differential gene expression analysis was performed to identify genes and pathways upregulated or downregulated upon Cep55 loss in the PTEN‐KD tumor context. Analyses were conducted both across the whole tissue section and within pathologist‐annotated lymphoma regions. Integrated datasets analyzed using AI‐ and machine learning‐based approaches, following published strategies for spatial omics data interpretation in cancer [27]. Low‑quality spots IN Visium dataset (fewer than 400 genes or over 30% hemoglobin content) and the P09 sample (69 spots versus an average above 220) were removed to prevent normalization artifacts and ensure reliable downstream analysis.

### 2.9 Immune Profiling, Genomics and Bioinformatics

Public TCGA RNA‐seq data (32 cancer types) were mined for CEP55 expression correlations with ∼1387 oncogenic signatures (cell cycle, mitosis, DNA repair, etc.) and immune cell infiltration scores (TIMER). Immune cell abundances were inferred by CIBERSORT and TISIDB, correlating CEP55 expression or copy‐number with 28 tumor‐infiltrating lymphocyte (TIL) types. TISIDB was further used to examine CEP55 associations with immunomodulators (checkpoint molecules, cytokines) and clinical outcomes across cancers. Differentially expressed mRNAs, proteins and phosphosites (Log2 fold change>0.5, *p* < 0.05) were subjected to Gene Ontology (GO) enrichment analysis to identify impacted pathways. All data analysis used R with appropriate packages; Pearson correlation and statistical significance were defined as *p* < 0.05, in proteomics pathway clusters *p* < 0.01. The integration was performed for enriched adhesion/integrin, immune and endosomal signature genes across samples. Mean normalized enrichment scores per signature module, heatmap of integrated pathways and normalized scores for immune modules are presented Cep55 expressing cells against the condition (Cep55 KO or CP‐DKD).

### 2.10 Statistical Analysis

Two‐tailed unpaired or paired Student's *t*‐test, one‐way or two‐way ANOVA with Bonferroni post hoc correction and log‐rank testing were performed where appropriate using Prism v9.0 (Graph Pad Software, La Jolla, CA, USA) and the P‐values were calculated as stated in the Figure legends. Statistical significance was noted in each Figure as follows: “no significance” (ns), *p* > 0.05; *p ≤ 0.05; **p ≤ 0.01, ***p ≤ 0.001, and ****p ≤ 0.0001. Mean ± SD: Mean and standard error of the mean were used to describe the variability within the sample in our analysis.

## 3 Results

### 3.1 CEP55 Is Broadly Overexpressed in human Cancers and Correlates With Poor Prognosis

Analysis of *CEP55* expression across the Cancer Genome Atlas (TCGA) dataset revealed that CEP55 is broadly overexpressed in a wide range of human malignancies, consistent with a previous report [28]. Pan‐cancer RNA‐seq analyses demonstrated significantly elevated CEP55 mRNA in many tumor types when compared to their corresponding normal tissues. Notably, high expression levels were observed in breast invasive carcinoma (BRCA), bladder urothelial carcinoma (BLCA), colon adenocarcinoma (COAD), glioblastoma multiforme (GBM), lung adenocarcinoma (LUAD), lung squamous cell carcinoma (LUSC), ovarian serous cystadenocarcinoma (OV), and pancreatic adenocarcinoma (PAAD) (Figure S1A). Clinically, elevated CEP55 expression was associated with more aggressive disease characteristics, including advanced tumor stage and grade, increased incidence of metastasis and significantly shorter overall survival (hazard ratios> 1; log‐rank *p* < 0.01) across multiple cancer types. Moreover, CEP55 expression increased progressively from stage I to IV and from low‐ to high‐grade tumors, most clearly in kidney renal papillary cell carcinoma (KIRP), kidney renal clear cell carcinoma (KIRC), lung adenocarcinoma (LUAD), and liver hepatocellular carcinoma (LIHC), and with tumor grade in brain lower grade glioma (LGG), uterine corpus endometrial carcinoma (UCEC), kidney renal clear cell carcinoma (KIRC), and liver hepatocellular carcinoma (LIHC). These associations were consistent across datasets and indicate that CEP55 upregulation is a strong marker of poor prognosis (Figure S1B and Sup material_1_Fig Aa).

To investigate the biological context that exacerbates the oncogenic role of CEP55, we analyzed CEP55 correlation with 1387 gene signatures representing oncogenic pathways and cancer hallmarks across 32 TCGA cancer types. These signatures encompassed critical processes such as cell cycle regulation, DNA replication/repair, immune signaling, apoptosis, oncogenic signaling, and metastasis‐related processes. Spearman correlation analysis across RNA‐seq datasets (n > 100 patients per cancer type) identified breast cancer, lung, sarcoma, skin cutaneous melanoma, and thymic tumors the strongest correlations between CEP55 expression and oncogenic pathway activation (Figure S1C and Sup material_2). Functionally, CEP55, known as a centrosomal protein and master regulator of cytokinesis, was most strongly associated with pathways related to mitotic spindle formation, microtubule organization, and cell division (Figure S1). Positively correlated pathways included cell cycle regulators (p53, RB, FOXM1), DNA damage response (ATM/ATR signaling, MRN complex), oncogenic signaling (MYC, PLK, Aurora kinases, p38 MAPK), immune pathways (IL‐6, IL‐2, TNF, MHC II), and apoptosis‐related programs (Table S1).

### 3.2 CEP55 Expression Is Linked to Immune Cell Infiltration and Epigenetic Modulation

We next examined CEP55 in the context of the tumor immune microenvironment (TIME), using data from CIBERSORT and TISIDB databases. High CEP55 expression positively correlated with the inferred abundance of several immune cell types, including activated memory CD4^+^ T cells, T follicular helper (T_fh) cells and Th2 cells (Figure S1D,E). Tumors with the highest immune correlation included cervical squamous cell carcinoma and endocervical adenocarcinoma (CESC), colon adenocarcinoma (COAD), head and neck squamous cell carcinoma (HNSC), kidney renal papillary cell carcinoma (KIRP), ovarian serous cystadenocarcinoma (OV) and thyroid carcinoma (THCA) (Figure S1D and Sup material_3). Analysis of CEP55 copy number variation and promoter methylation further revealed that CEP55 copy number gain was associated with modest immune cell changes; however, CEP55 promoter hypermethylation consistently correlated with reduced lymphocyte infiltration (Sup material_1 Fig b). This suggests that epigenetic silencing of CEP55 may suppress immune infiltration and contribute to immune evasion mechanisms in tumors.

CEP55 expression aligns with immune checkpoints and immune evasion signatures. Interestingly, CEP55 expression also correlated with the expression of multiple immune checkpoint molecules. Specifically, some of the tumor types with high CEP55 levels exhibited elevated expression of inhibitory checkpoint genes such as *TIGIT*, *LAG3*, *CTLA4*, *PDCD1* and, with a weaker but consistent co‐occurrence, with immunostimulatory ligands such as *CD80* and *CD86* (Figure S1E). Notably, CEP55‐high tumors displayed decreased *MHC‐I* gene expression, suggesting impaired antigen presentation and a potential mechanism for immune evasion (Sup material_1 Fig c). Despite this reduction in *MHC‐I*, expression of *TAP1* and *TAP2*, key genes in the antigen‐processing machinery, remained positively correlated with CEP55, indicating that while antigen processing is retained, surface presentation may be limited. Furthermore, CEP55 expression demonstrated an inverse correlation with the immunosuppressive cytokine TGF‐β1 and a positive correlation with pro‐inflammatory chemokine CXCL8 (IL‐8). In contrast, CCL14, a chemokine associated with monocyte recruitment, was lowest in CEP55‐high tumors (Sup material_1 Fig c‐d).

These findings support the existence of a complex immune contexture in CEP55‐high tumors: while enriched for immune infiltrates, these tumors limit anti‐tumor responses through immune checkpoint upregulation and reduced antigen visibility via low MHC‐I. Furthermore, in thyroid, colon, ovarian, and cervical cancers, elevated CEP55 coincided with a Th2‐skewed immune response and was lowest in the C5 (immune‐cold) subtype (Sup material_1 Fig e). In contrast, in breast cancer, CEP55 was most highly expressed in the C2 subtype characterized by an IFN‐γ–dominant immune response, and again lowest in the C5 subtype (Sup material_1 Fig e). These data suggest that CEP55 upregulation not only promotes proliferation and cell‐cycle progression but also shapes an immunosuppressive tumor microenvironment linked to adverse clinical outcomes.

### 3.3 Proteomic Profiling of Downstream Effects Upon Cep55 Loss in Untransformed and Early‐Stage Transformed Mouse Embryonic Fibroblasts (MEFs)

To assess whether CEP55 ablation impacts the cell proteome content and quality, we performed mass spectrometry‐ based proteomic analysis using primary or transformed MEFs, under Cep55 wildtype (Wt) or knockout (KO) conditions. Primary untransformed MEFs (pMEFs) were immortalized using the SV40 large T antigen, to mimic early transformation, or the adenoviral E1A/Ras^V12^ vector to mimic a tumorigenic stage, E1A/Ras [9]. The workflow of proteomics and phosphoproteomics is illustrated in Figure 1A.

**FIGURE 1:**
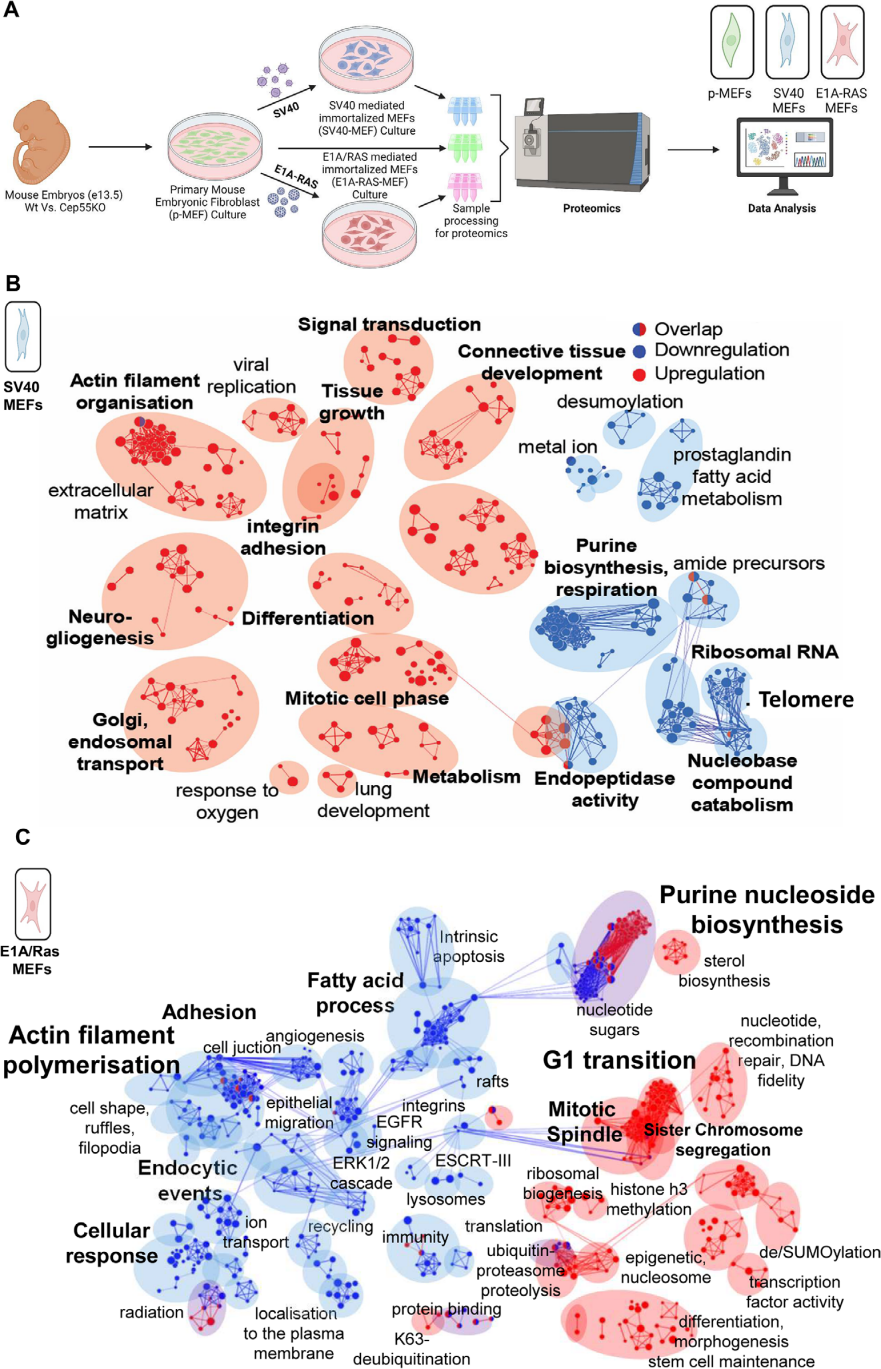
Proteomic profiling reveals distinct stage‐specific molecular alterations upon Cep55 loss. (A) A schematic overview of the experimental workflow. Mouse embryonic fibroblasts (MEFs) derived from wild‐type (Wt) and *Cep55* knockout (KO) embryos were cultured as primary (p‐MEFs), SV40‐immortalized, or E1A/Ras‐transformed lines and subjected to total and phospho‐proteomic analyses. LC‐MS/MS analyses were performed using independently cultured and processed biological replicates (n ≥ 3 per condition). Pathway enrichment analyses were conducted on significantly altered proteins or phosphosites defined by |log_2_ fold change| > 0.5, *p* < 0.05, and FDR < 0.05. Data are presented as mean ± SD unless otherwise indicated. (B,C) Network clustering of significantly enriched biological processes altered in Cep55 KO versus Wt MEFs at progressive stages of transformation: (B) SV40‐immortalized and (C) E1A/Ras‐transformed MEFs. Upregulated pathways are shown in red, downregulated pathways in blue, and overlapping processes in purple. Key functional modules include cytoskeletal organization, integrin adhesion, Golgi/endosomal transport, metabolism, and mitotic spindle regulation, indicating stage‐specific rewiring of signaling and cellular architecture following Cep55 loss.

We first focused on the pMEF and SV40 conditions, identifying ∼2000 total proteins and 300–400 phosphoproteins. These analyses revealed significantly up‐ or downregulated proteins (q<0.05) upon Cep55 loss relative to Wt (Figure S2A,B). The correlation between pMEFs‐Wt and ‐KO proteome profiles (Spearman r ≈ 0.74) suggests that the lack of Cep55 causes protein expression changes, while normalized intensities across replicates indicate unchanged global proteome amount (Figure S2C,D **)**. The cluster maps of enriched pathways in SV40‐MEFs (early transformation stage (Figure 1B) and E1A/Ras (tumorigenic stage, Figure 1C) suggested rewiring of several unexpected and separate biological processes in KOs.

Downregulated processes in SV40‐MEFs included ribosomal biogenesis, prostaglandin signaling, nucleotide metabolism, and fatty acid biosynthesis (blue, Figure 1B). Analysis of individual biological and molecular pathways in both pMEFs (untransformed) and SV40‐MEFs revealed that significantly differentially downregulated proteins (log_2_ fold change > 0.5, p < 0.05) are involved in RNA processing, mRNA translation, and ribosomal biogenesis in Cep55‐KO cells. Notably, ribosomal proteins such as Rpl24 and Rps23, along with several DEAD‐box helicases implicated in ribosomal biogenesis and translational control, were significantly reduced in both KO conditions (Figures S2E–G and S3A–D). While some of these effects are likely indirect, they may also be linked to altered centrosome architecture. The centrosome and midbody have been shown to spatially localize specific RNAs (e.g. active translation of CHMP4B at midbody, a CEP55‐linked protein), and to associate with spliceosomal components, often in conjunction with ribosomal proteins, ribonucleoprotein complexes, and elements of translational control [29, 30, 31].

Interestingly, SV40‐MEF KO cells exhibited upregulation of pathways involved in actin filament organization, extracellular matrix (ECM) remodeling, integrin‐mediated adhesion, connective tissue development, and neurogliogenesis (red, Figure 1B). Notably, the upregulated transforming growth factor beta (TGF‐β) pathway driven by proteins such as cytokine TGF‐β receptor II (Tgfbr2) and Cd109 is consistent with the observed inverse correlation between CEP55 and TGF‐β expression (Sup material_1 Fig c,d). The enriched upregulated clusters in SV40‐MEF KO (Figure 1B), and their pathways, summarized in Table S2, collectively suggest that Cep55 loss during early transformation modulates pathways involved in tissue growth, cell behavior, differentiation, and immune regulation.

Remarkably, Cep55‐KO in both pMEFs and SV40‐MEFs resulted in significant upregulation of cell‐substrate adhesion and ECM pathways (Figure S3E–G), in contrast to downregulation observed in the tumorigenic E1A/Ras stage (Figure 1C). Key drivers of these processes included laminin and RGD‐receptor integrins ItgaV, Itgb1 and especially in SV40‐MEF‐KO Itga5 (Figure S3E,F), along with their ECM‐binding partner Thrombospondin 1 (Thbs1), and several collagens such as Col3a1 and Col1a1 (Figure S3G,H). Upregulation of integrins (Figure S3E,F) and collagens (Figure S3G,H) suggested increased cell‐cell and connective tissue interactions.

Both pMEF and SV40‐MEF KO conditions also exhibited increased expression of variable actin‐phospholipid membrane‐binding proteins (Figure S3C,D), again in contrast to the tumorigenic E1A/Ras condition (Figure 1C). Notable examples include epidermal growth factor receptor (Egfr) and myristoylated alanine‐rich C‐kinase substrate (Marcks) in pMEF‐KO, as well as Bridging Integrator 1 (Bin1) and cell adhesion molecule Kirrel1 in SV40‐KO. Many of these proteins are either regulators or targets of endocytic processes. CEP55 interacts with the ESCRT proteins TSG101 and ALIX at the midbody during cytokinesis [4, 32], where ESCRT‐I/II complexes recruit the ESCRT‐III subunit CHMP4B to facilitate abscission [3]. Beyond their role in cytokinesis, ESCRT complexes are central to the endosomal sorting and degradation of ubiquitinated membrane proteins, including integrins, and are implicated in a wide range of cellular processes, from lipid, fatty acid, and amino acid metabolism to signaling, genome maintenance, and membrane repair [33, 34]. Proteomic heatmaps revealed reconfiguration of the endosomal and lysosomal adaptor and regulator proteins in Cep55‐KO pMEF and SV40‐MEF cells, including altered expression of ESCRT‐0 to ‐III subunits such as Stam2 and Hrs (Hgs) (Figure S4A–C). Notably, Chmp4b (ESCRT‐III) was downregulated, and regulators of integrin trafficking, such as Ptpn23 (Hd‐ptp) [23], showed differential expression. In summary, proteomic profiling of Cep55‐KO primary (pMEFs) and early‐transformed (SV40‐MEFs) suggests that reprogramming of the endosomal pathway may underlie the elevated expression of certain signaling and adhesion receptors (e.g. integrins) and contribute to the observed metabolic, developmental and differentiation‐related processes (Figure S2E–G).

### 3.4 CEP55 Loss Drives Distinct Proteomic and Phosphoproteomic Changes in Tumorigenic E1A/Ras MEFs

We next focused on the impact of CEP55 loss at the tumorigenic stage using E1A/Ras‐transformed MEFs, which model oncogenic transformation. Following the workflow in Figure 1A, we analyzed ∼5000 total proteins and phosphopeptides with ∼500 phosphosites. Compared to primary (pMEFs) and early‐transformed (SV40‐MEFs) (Figure 1B, Figures S2E,F and S3A–D), E1A/Ras‐transformed MEFs exhibited substantial differences in enriched clusters and biological and molecular pathways (Figure 1C, Figures S2G and S5A—D). These upregulated and downregulated pathways and clusters are summarized in Table 1. Strikingly, Cep55‐E1A/Ras KO cells exhibited downregulation of pathways related to cell adhesion, endocytosis and oxidative stress response, which contrasted with upregulation of these pathways observed in pMEF and SV40‐KO cells (Figure 1C, Figure S5A,B). Processes such as integrin‐mediated signaling, focal adhesion and integrin binding were decreased, driven by proteins such as integrins ItgaV, Itgb3, Itgb5, and focal adhesion protein tyrosine kinase 2 (Fak; Ptk2) and late‐endosomal tetraspanin Cd63 (Figures S2G and S5C,D). While ECM binding components were decreased, the amount of collagen remained unchanged in E1A/Ras KO (Figure S5E).

**TABLE 1:** Differentially Up and Downregulated pathways in Cep55 E1A‐Ras‐MEFS.

Cluster	Pathways Upregulated E1A/RAS‐MEFs KO/Wt
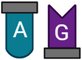 **Purine biosynthesis**	Purine ribonucleotide biosynthetic process Cellular respiration, Oxidative phosphorylation ATP biosynthetic process
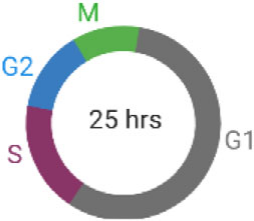 **G1 transition cell cycle**	Negative regulation of mitotic cell cycle phase transition Cell cycle G1/S phase transition
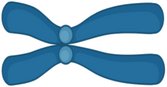 **Sister chromosome Separation**	Positive regulation of chromosome segregation Mitotic spindle midzone assembly Centrosome cycle
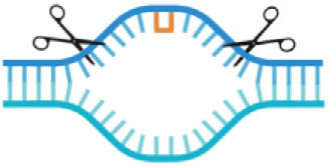 **DNA repair,** 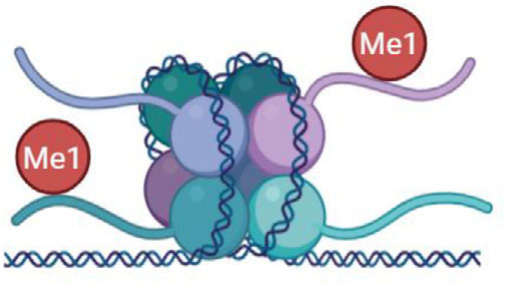 **Epigenetics** 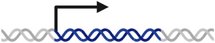 **Transcription factors**	Negative regulation of DNA recombination DNA‐templated DNA replication maintenance of fidelity Epigenetic regulation of gene expression Histone methylation Histone H2B ubiquitination Regulation of protein sumoylation Regulation of DNA‐binding transcription factor activity
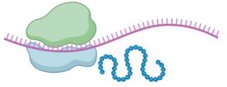 **mRNA translation**	Ribosome biogenesis Negative regulation of mRNA metabolic process Negative regulation of translation
**Cluster**	**Pathways downregulated E1A/RAS‐MEFs KO/Wt**
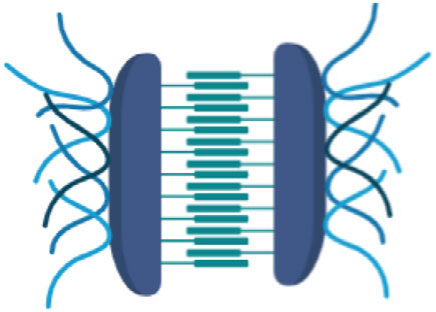 **Adhesion**	Positive regulation of cell‐matrix adhesion, Focal adhesion assembly Positive regulation of epithelial cell migration (e.g. integrin signaling); Adhesion spreading substrate Positive regulation of smooth muscle cell migration
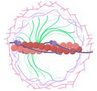 **Actin Filament polymerization or stabilization**	Actin filament organization Actin polymerization or depolymerization Ruffle organization
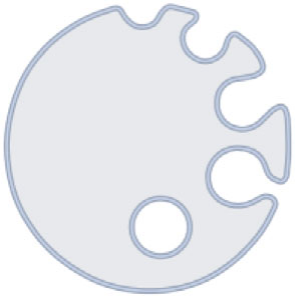 **Endocytic events**	Regulation of endocytosis, recycling, phagocytosis, internalization, autophagy, lysosomes ERK1/ERK2 cascade Endosomal transport Epidermal growth factor receptor signaling pathway
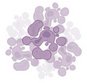 **Apoptosis**	Regulation of apoptotic signaling pathway
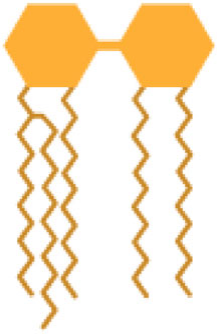 **Fatty acids, lipids**	Regulation of lipid metabolic process Fatty acid biosynthetic process Prostaglandin biosynthetic process Lipid transport Plasma membrane raft assembly
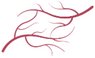 **Angiogenesis**	Regulation of angiogenesis Positive regulation of vasculature development
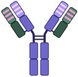 **Immunity**	Regulation of leukocyte migration, Macrophage chemotaxis Negative regulation of immune system process
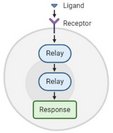 **Cell responses**	Response to hypoxia Reactive nitrogen species metabolic process Reactive oxygen species metabolic process Regulation of monoatomic ion transmembrane transport

In contrast, the upregulated pathways in E1A/Ras‐CEP55 KO cells involved chromatin remodeling, nucleosomal DNA binding (e.g., histones h1‐0, h1‐1), and regulatory proteins including histone methyltransferase SET Domain Containing 2 (Setd2), serine/threonine‐protein kinase tousled‐like 2 (Tlk2), and HAUS‐family spindle assembly proteins. These findings suggest a shift in epigenetic regulation, DNA damage response, and mitotic control (Figures S2G and S5A,B). Phosphoproteomic analysis further revealed increased phosphorylation of proteins involved in chromatin dynamics, cytokinesis, and mRNA processing, while phosphorylation was decreased in pathways related to actin organization, transcription elongation, and translation regulation (Figure S5C,D). As expected, the downregulation of Akt1 phosphorylation (Ser^473^) and mitosis‐specific Cep55 (Ser^426^) phosphorylation was observed. Phosphorylation was notably reduced in several sites of Bridging integrator 1 (Bin1), a tumor suppressor that binds Myc, and Fibroblast growth factor receptor substrate 2 (Frs2), a key signal‐transducing adaptor protein involved in the MAPK/ERK, PI3K/AKT/mTOR signaling [35]. Specifically downregulated Frs2 phosphosites included (T132; T135; T137, S211, S365) along with phosphosites of cytoskeletal protein Pdlim2 and vesicular trafficking and cilium assembly protein Rab3ip (Figure S5E and Table S3). In contrast, increased phosphorylation was observed in the Breakpoint cluster region kinase (Bcr; at multiple sites including S461 and S464) and DNA methyltransferase 1 (Dnmt1) in E1A/Ras KO MEFs. Other proteins with significantly upregulated phosphorylation included microtubule‐associated proteins (Kif23), centrosome‐endosomal proteins (Cc2d1a) and signaling proteins (Pak1) (Figure S5E and Table S3). Notably, some phosphosites involved in chromatin binding (Hmga1), DNA repair (Tnks1bp1), mRNA splicing (Srrm1 and 2), stress‐responsive translation (Ubap2l), cell shape and motility (Myh9, Map7d1) were consistently up‐ or downregulated across pMEF, SV40, and E1A/Ras KO MEFs (Figure S5H). Phospholipase A2 IVA (Pla2g4a; cPLA2α) was downregulated at both total protein and phosphorylation in all KO conditions (Figure S5H), correlating with suppression of the prostaglandin/fatty acid metabolism pathway (Figure S5G).

Together, these data demonstrate that genetic ablation of Cep55 in an oncogenic background lead to distinct proteomics and phosphoproteomic alterations in comparison to primary and early transformed MEFs. These changes likely reflect the altered signaling and metabolic landscape characteristic of fully transformed cells.

### 3.5 CEP55 Expression Modulates Integrin Receptor Endocytosis, Signaling, and Cell Migration

To functionally validate Cep55 KO‐associated alterations in early transformed (SV40) MEFs, we analyzed the effect of Cep55 loss on integrin receptors, a connection not yet explored. Several RGD‐motif‐binding integrin receptors, such as integrin α5β1, undergo enhanced internalization and recycling in oncogenic cells, a process linked to increased cell migration, cancer progression, and metastasis. Tight regulation of dynamics in endocytosis, actin cytoskeleton, ligand binding, ECM interaction and integrin clustering is essential to coordinate integrin activation [23, 36].

To assess integrin trafficking, we leveraged the pH‐dependent nature of different endosomal compartments to quantitatively track cargo at the single‐vesicle and single cell level using pH‐sensitive, fluorescence‐conjugated probes combined with fluorescence microscopy [22, 23, 37]. Transferrin receptor (TfR) served as a control for fast, ubiquitin‐independent endocytosis and dextran (Dx) for non‐specific endocytosis. Labeled integrin α5β1 (IntαR) endocytosis in early transformed SV40 Wt (+/+) MEFs revealed that internalized integrins localize to compartments with overlapping recycling (∼pH 6.3) and early endosomal (pH 6.0–6.4) pH profiles, consistent with signaling‐competent integrin trafficking dynamics (Figure 2A,B).

**FIGURE 2:**
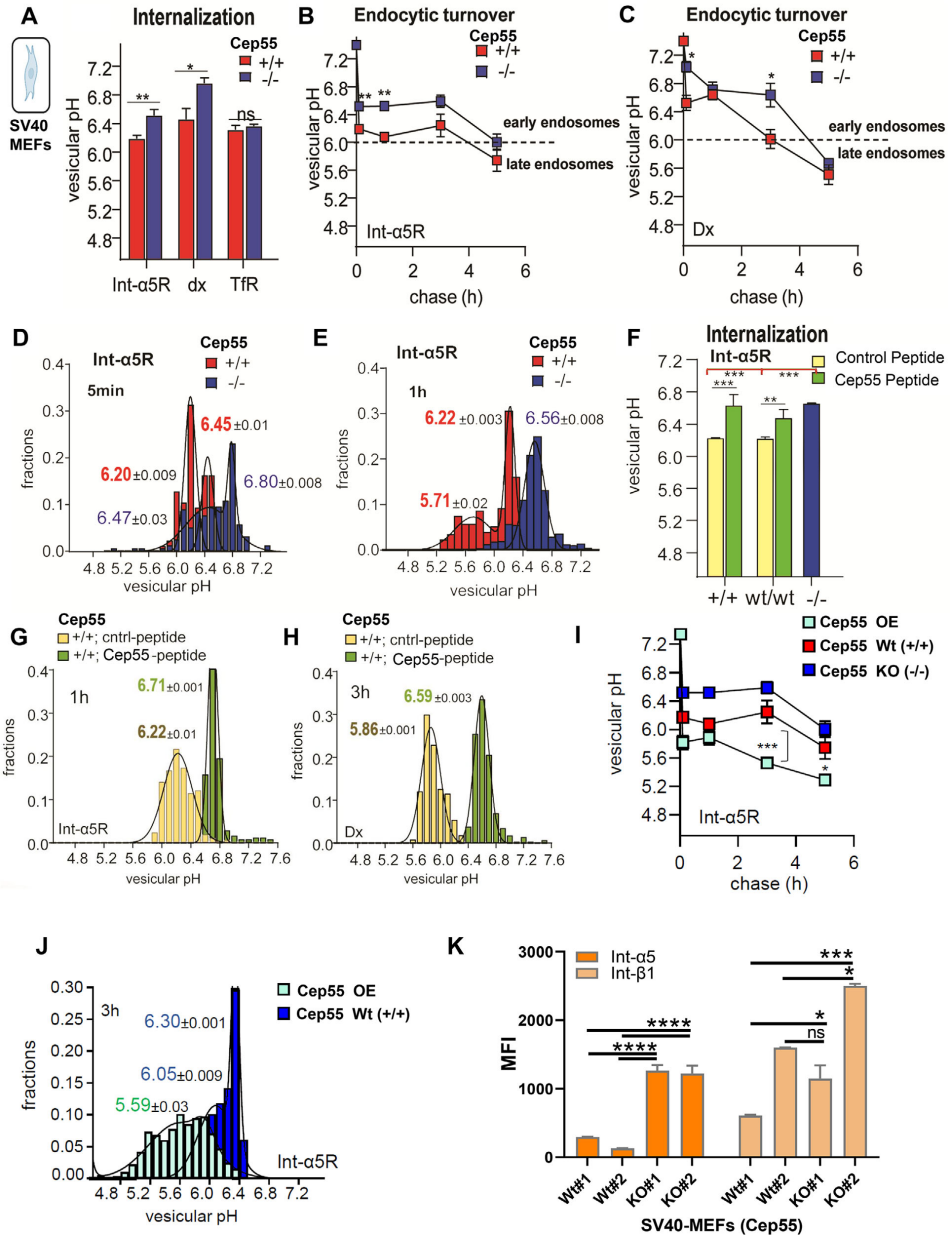
Cep55 is a novel regulator of integrin α5β1 endocytosis and trafficking. (A–C) Live‐cell single‐vesicle pH microscopy and analysis of integrin α5β1 endocytosis kinetics. Typical vesicular pH for sub‐plasma membrane endosomes progresses from the near‐neutral pH to recycling endosome ∼pH 6.3, early endosome pH 6.0–6.4, late endosomes/multivesicular body pH 6.0–5.5 to the acidic lysosomes pH<5.5. Cells were incubated with pH‐sensitive fluorophore‐labeled cargo for indicated time points to assess the endosomal turnover in Cep55 KO (−/−) and Wt (+/+) SV40 MEFs. Transferrin receptor (TfR) was used as a marker for fast, ubiquitin‐independent endocytosis and dextran (Dx) served as a control for non‐specific endocytosis. (D,E) Representative histograms showing vesicular pH distribution for internalized integrin α5β1. (F–H) Endocytic pH analysis following treatment of cells with a Cep55‐derived peptide that disrupts the Cep55–Alix interaction, and effects on integrin α5β1 (F,G) and Dx (H) trafficking mimic the Cep55 KO phenotype. (I) Live‐cell single‐vesicle pH microscopy and analysis of integrin α5β1 endocytosis kinetics. The endosomal turnover was measured in Cep55 overexpression (Cep55 OE), Cep55 Wt (+/+) and Cep55 KO (−/−) MEFs. (J) Representative histogram showing vesicular pH distribution for internalized integrin α5β1 for Cep55 OE and Wt. (K) Flow cytometry (FACS) analysis of cell surface expression of integrin α5 and β1. MFI: mean fluorescence intensity. A–C, F and I: Data shown as means ± SD, n ≥ 3. *p*‐value: ns; non‐significant, *p < 0.05, **p < 0.01, ***p < 0.001, ****p < 0.0001. D,E, G,H: Analysis from n >300 vesicles, >50 cells.

Cep55 loss significantly delayed integrin α5β1 internalization and its targeting to early and late endosomes (pH < 6) (Figure 2A,B). In Cep55 KO (‐/‐) cells, integrin α5β1 exhibited prolonged residence in sub‐plasma membrane vesicles (pH > 6.5), whereas in Wt (+/+) cells, the receptor localized to recycling compartments (pH 6.3) and late endosomes (pH 5.1) (Figure 2D,E). Immunofluorescence microscopy of internalized integrin α5β1 receptor confirmed its colocalization with the early endosomal marker EEA1 in Wt cells, but not in Cep55 KO cells (Figure S6A). Furthermore, dextran internalization and endocytosis were delayed in Cep55 KO (‐/‐) cells (Figure 2A,C), suggesting that Cep55 alters endosomal organelle structure and dynamics.

To test whether disrupting the interaction between Cep55 and the ESCRT adaptor Alix (Pdcd6ip), critical for abscission, affects integrin trafficking, we used an ALIX‐peptide (GPPSLNY) [4] that blocks the Cep55–Alix binding site interaction and a control peptide with alanine substitution (GPASLNA) in two SV40‐transformed wild‐type (Wt/Wt, +/+) cell lines. Endocytic pH analysis revealed that this Cep55‐Alix‐peptide mimicked the KO phenotype, significantly delaying integrin α5β1 trafficking and resulting in sub‐plasma membrane vesicle localization (pH 6.6–6.7) (Figure 2F,G), also delaying dextran‐positive vesicles to recycling compartments (∼pH 6.3) after 3 h instead of late endosomes (Figure 2H). To support the specificity of the Cep55 phenotype in the graded, dose‐dependent manner, we included CEP55‐overexpressing MEFs as a reciprocal rescue‐like condition. CEP55 overexpression accelerated the shift of internalized integrin α5β1 toward late endosomal compartments, whereas Cep55 KO delayed this transition and retained integrin α5β1 in early endosomal vesicles. These gain‐ and loss‐of‐function data together indicate that CEP55 dosage regulates integrin α5β1 endosomal trafficking (Figure 2I,J).

FACS analysis confirmed increased cell surface expression of integrin α5 and β1 in SV40 Cep55‐KO MEFs, consistent with proteomics findings (Figure 2K and Figure S6B). Since integrin endocytosis was impaired in SV40 KO cells, suggesting defective integrin activation, we next assessed downstream signaling. In SV40 KO cells, phosphorylated Akt (Ser^473^) and phosphorylated ERK1/2 (Phospho‐p44/42 MAPK, Thr^202^/Tyr^204^), both key β1‐integrin signaling effectors, [38] were downregulated in a Cep55 expression‐dependent manner while the total proteins and upstream mediators remained unchanged (Figure S6C). Similar trends were evident in E1A/Ras‐transformed MEFs, where despite comparable KRAS expression across conditions, Cep55 KO led to decreased levels of phosphorylated ERK1/2, integrin β5, phosphorylated focal adhesion kinase (pFAK = pPTK2), phosphorylated Paxillin, and RHOA (Figure S6D). Notably, in E1A/Ras MEFs, Cep55 deletion also reduced expression of integrin α5, a subunit frequently upregulated in oncogenic contexts. These findings indicate that Cep55 is critical for maintaining integrin activation and downstream signaling required for oncogenic signaling in transformed cells.

To examine the functional consequences of Cep55 loss on cell motility, we performed time‐lapse single‐cell tracking using the HoloMonitor live‐cell imaging. Cep55‐KO cells exhibited significantly reduced motility, with approximately 20%–30% lower average migration and total distance traveled per cell compared to wild‐type controls (Figure S6E). These findings indicate that Cep55 loss primarily impairs migration velocity, likely because of disrupted integrin trafficking and signaling. Taken together with our previous observations, the data show that Cep55‐KO disrupts integrin endocytosis, recycling, and activation, leading to attenuated focal adhesion signaling as evidenced by decreased phosphorylation of FAK and paxillin (Figure S6D). Concurrent suppression of RHOA and pERK1/2 signaling, likely contributes to altered cytoskeletal organization and reduced migratory capacity. These data support a model in which Cep55 is essential for sustaining integrin‐dependent adhesion and signaling required for efficient cell migration.

To explore the clinical relevance of these findings, we queried the cBioPortal and The Cancer Genome Atlas (TCGA) datasets to assess whether CEP55 expression correlates with integrin signaling components in human cancers, particularly in breast cancer, where integrin signaling is a well‐established driver of disease progression. We analyzed a cohort of 963 patients with invasive breast carcinoma, focusing on mRNA expression and copy number alterations for genes including CEP55, integrins αV, α5, β1, fibronectin (FN1), and FAK (PTK2). Our analysis revealed strong co‐occurrence and positive correlation between CEP55 expression and the expression of integrins β1, αV, α5, fibronectin, and FAK (Figure S6F and Table S4). These results suggest that the relationship between CEP55 and integrin‐mediated signaling observed in our experimental models may be conserved in human cancers, supporting a broader role for CEP55 in regulating adhesion and signaling networks that drive tumor progression.

### 3.6 Cep55 Knockout Impairs Cell Migration, Transformation and Invasive Growth In Vitro

To evaluate the role of Cep55 in integrin‐regulated cellular behaviors, we assessed motility, migration, and invasion in oncogenic E1A/Ras MEFs. In wound‐healing assays, Cep55‐KO (‐/‐) cells exhibited significantly impaired migration, closing approximately 40% less wound area than Wt (+/+) cells after 50 h (Figure 3A). In our custom 3D spheroid invasion assays using PIC‐GFOGER hydrogel matrices, Cep55‐KO cells showed reduced invasion areas at 24‐ and 48‐h post‐embedding compared to oncogenic Wt cells (Figure 3B). We examined several hydrogel conditions for the 3D spheroid invasion assay and tailored the hydrogel physicochemical properties and microenvironment to support optimal spheroid invasion conditions. We found that the combined matrix stiffness of 330 Pa, 1% GFOGER ligand density and strain stiffening onset of 37 Pa provided a scaffold that supported spheroid stability, growth, and cell migration through the hydrogel microenvironment. (Figure 3C). Moreover, a collagen adhesion assay revealed significantly reduced adhesion of KO MEFs (Figure 3D). Collectively, these findings confirm that Cep55 supports integrin‐driven migration, adhesion and invasive capacity in oncogenic MEFs.

**FIGURE 3:**
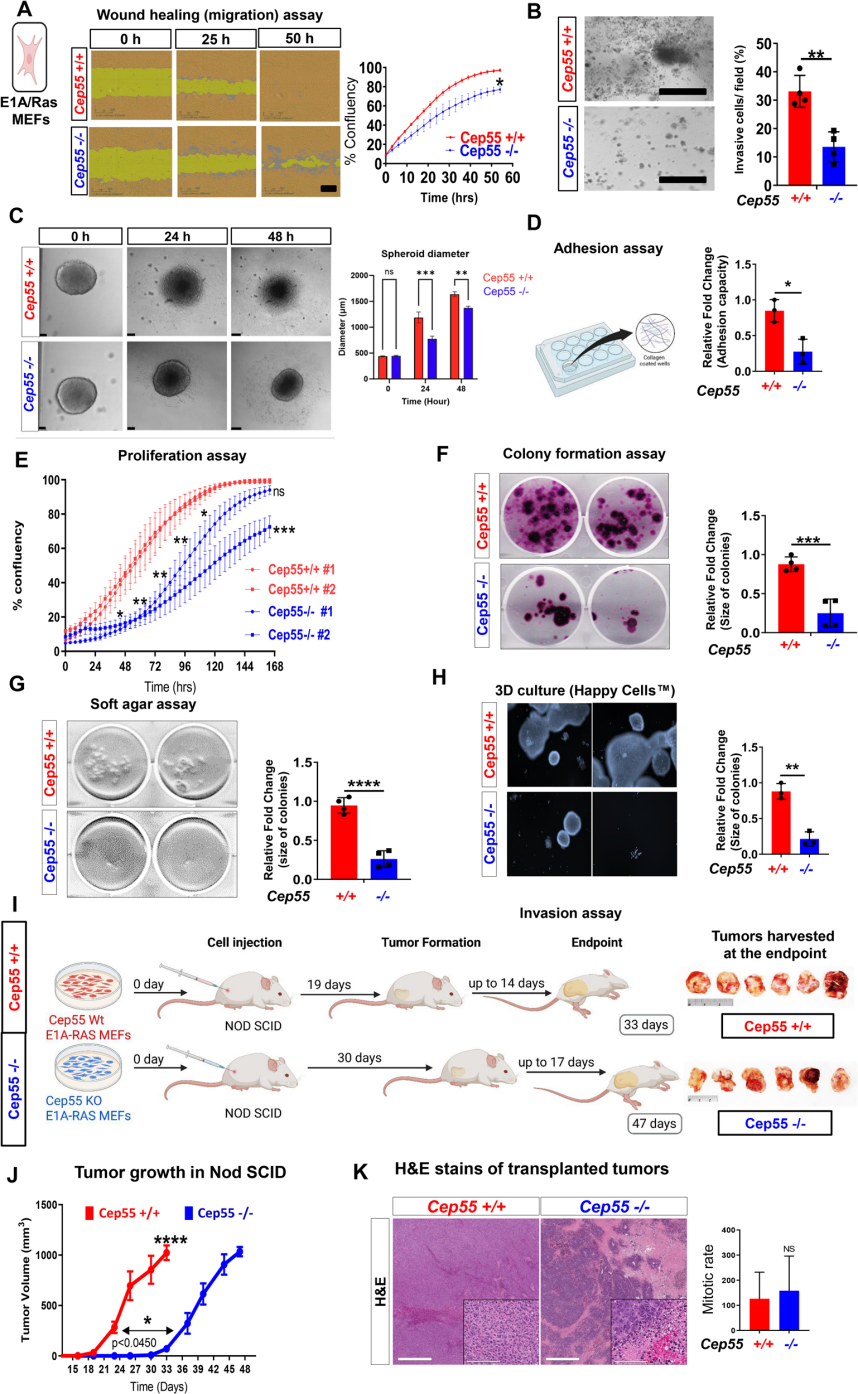
Cep55 is essential for cell proliferation, migration, invasion, and tumorigenic potential. (A) Wound‐healing (migration) assay comparing E1A/Ras‐transformed MEFs from Cep55 wild‐type (Wt) and knockout (KO) lines, showing significantly reduced migratory capacity in Cep55‐deficient cells. For in vitro studies, experiments were performed in duplicate for two biological replicates and repeated in at least two independent experiments unless otherwise indicated. Data represent mean ± SD; two independent experiments; ns; non‐significant, *p < 0.05, **p < 0.01, ***p < 0.001, ****p < 0.0001. (B) Transwell invasion assay demonstrating impaired invasive ability in Cep55 KO cells. Bar graph represents relative fold change in invaded cell area. (C) Spheroid invasion assay using a collagen‐mimicking polyisocyanopeptide (PIC)‐based hydrogel showing attenuated spheroid expansion in Cep55 KO spheroids over time. (D) Cell adhesion assay on collagen‐coated plates reveals reduced adherence in the absence of Cep55, average of three independent experiments. (E) Proliferation kinetics measured by IncuCyte live‐cell imaging showing decreased confluency in Cep55 KO clones over 144 h. (F) Colony formation (clonogenic) assay showing reduced long‐term survival and clonogenic potential in Cep55 KO MEFs. (G) Soft agar assay demonstrates diminished anchorage‐independent growth in CEP55‐deficient cells. (H) 3D spheroid culture using the Happy Cells system confirms reduced spheroid formation capacity in Cep55‐KO lines. Average of three independent experiments. (I) Schematic of in vivo tumor formation assay following subcutaneous injection of E1A/Ras MEFs (Cep55 Wt and KO) into NOD/SCID mice. (J) Tumor growth kinetics showing delayed onset and significantly reduced tumor volume in Cep55 KO xenografts. (K) Representative hematoxylin and eosin (H&E)‐stained tumor sections show no reduced mitotic index in Cep55 KO tumors. Data represent mean ± SD (n = 6 mice per group; two independent experiments; Student's *t*‐test, ****p < 0.0001).

To assess whether Cep55 loss also affects tumorigenic characteristics, we next analyzed proliferative capacity and clonogenicity potential in E1A/ Ras MEFs, Real‐time live‐cell imaging (IncuCyte) revealed that Cep55‐KO^–/–^ MEFs proliferated at ∼50% the rate of Cep55‐Wt^+/+^ cells, with noticeable growth defects emerging by 48–72 h and a ∼2‐fold reduction in confluence observed by days 5–7 (*p* < 0.001) (Figure 3E). Clonogenicity was also significantly compromised in Cep55‐KO (‐/‐) MEFs compared to their Wt (+/+) counterparts, as evidenced by diminished colony formation in both 2D and 3D assays (Figure 3F). In soft agar, Cep55‐KO MEFs formed ∼70% fewer colonies, which were markedly smaller, typically less than one‐third the size of Wt colonies (Figure 3G). To further assess 3D growth in a suspension environment, we used Happy Cell Advanced Suspension Medium (HCM), a low‐viscosity liquid reagent matrix that enables spheroid formation from single cells [39, 40]. Similarly, in Happy Cell matrix, Cep55 KO E1A/Ras MEFs yielded dramatically fewer and smaller spheroids compared to Wt (Figure 3H). These results indicate that Cep55 is essential for anchorage‐independent growth and survival under non‐adherent conditions, underscoring its role in supporting anoikis resistance and tumorigenic potential.

### 3.7 Loss of CEP55 Delays Tumor Growth and Progression In Vivo (Cell Transplant Model)

Our in vitro findings demonstrate that Cep55 deficiency impairs multiple oncogenic behaviors in MEFs, including integrin‐mediated AKT and FAK signaling, adhesion, invasion, and anchorage‐independent growth. To determine whether these impairments translate to reduced tumorigenicity in vivo, we employed a cell transplant model. E1A/Ras‐transformed MEFs (either Cep55‐Wt (+/+) or Cep55‐KO (‐/‐) were injected subcutaneously into 8–10‐week‐old immunodeficient NOD/Scid mice (n  =  6 per group All mice injected with Cep55 Wt (+/+) MEFs developed palpable tumors by ∼19 days post‐injection. In contrast, mice injected with Cep55 KO (‐/‐) MEF developed palpable tumors only after ∼30 days (*p* < 0.04), a greater than 50% delay in tumor onset (Figure 3I). Moreover, once tumors were established, those derived from Cep55‐KO cells grew more slowly, reaching the experimental endpoint (1000 mm^3^) by day 47 on average, compared to day 33 for Wt tumors (*p* < 0.01). This delay in tumor progression was reflected in overall tumor growth kinetics (Figure 3I).

Histological examination of H&E‐stained sections of endpoint tumors revealed large necrotic regions in Cep55‐KO tumors, whereas WT tumors were more uniformly viable (Figure 3K, right panel). Interestingly, the mitotic index (mitoses per high‐power field) was not significantly different between WT and KO tumors (Figure 3K, left panel), suggesting that proliferation of the viable tumor cells in vivo was similar at endpoint (tumors harvested at different endpoints). However, the greater necrosis in KO tumors may reflect more cell death and possibly impaired vascularization or nutrient supply.

Together, these data demonstrate that Cep55 loss significantly delays tumor initiation and slows tumor progression in a transplant setting, consistent with the in vitro findings of impaired growth and survival. Combined with our unbiased proteomic data and bioinformatic analyses of human cancer databases, these findings highlight a critical and previously underappreciated role for Cep55 in regulating cell adhesion and integrin signaling via the endocytic and ESCRT pathway. Loss of Cep55 impairs integrin activation and downstream oncogenic signaling, culminating in suppressed tumor growth and invasive capacity. These results suggest that CEP55 may represent a novel target for therapeutic intervention in integrin‐driven malignancies.

### 3.8 A Novel Mouse Model of Inducible Cep55 cKO Marked Cep55 as Dispensable for Normal Adult Viability

Building on the anti‐tumor effect of Cep55 loss in our transplant model, we next employed a genetically engineered mouse model (GEMM) to further investigate its role in tumorigenesis in vivo. Our previously reported constitutive Cep55 knockout mouse was perinatal lethal, largely due to neurodevelopmental defects during embryogenesis, consistent with the known essential role of Cep55 during early development [9]. To overcome this limitation, we generated an inducible Cep55 conditional knockout (cKO) in adult mice using a knockout‐first allele amenable to the Cre‐LoxP system (Figure S7A). Through a breeding strategy (Figure S7B), we crossed Cep55^Fl/Fl^ mice with Rosa26‐CreERT2 mice to obtain Rosa26‐CreERT2^+;^ Cep55^Fl/Fl^ animals. In this system, Cre recombinase is expressed ubiquitously but remains inactive until tamoxifen administration, enabling controlled Cep55 deletion in adult tissues. To assess Cep55 expression, we first analyzed publicly available murine RNA‐Seq Cap Analysis of Gene Expression (CAGE) datasets, which revealed high *Cep55* RNA expression levels in testis and moderate levels in spleen, lung and intestine in adult mice (Figure S7C). We confirmed these findings at the protein level using immunoblotting in 8‐week‐old mice. The highest expression of Cep55 was observed in testis, with moderate expression in the spleen and intestines, and undetectable levels in the other tissues analyzed (Figure S7D **)**. Efficient CEP55 protein depletion following tamoxifen administration was confirmed in the spleen (Figure S7E). To further validate the model in vitro, we isolated MEFs from Rosa26‐CreERT2^+;^ Cep55^Fl/Fl^ embryos and induced knockout using 1 µg 4‐hydroxytamoxifen (4OHT) for 72 h. This induced robust Cep55 loss and significantly impaired cell proliferation, replicating the phenotype seen in constitutive Cep55 knockout MEFs (Figure S7F).

Next, we evaluated the physiological impact of Cep55 deletion in vivo in adult mice. 8‐week‐old Rosa26‐Cre^ERT2^; Cep55^Fl/Fl^ mice were treated with tamoxifen (1 mg) to induce global Cep55 knockout (n = 8), alongside littermate controls (Cre+ PBS injected, or Cre‐ Cep55^Fl/Fl^ tamoxifen‐treated; n = 6). Remarkably, all Cep55‐cKO mice remained viable, healthy (weight and appearance) and behaviorally normal for at least 25 weeks post‐induction, with no overt phenotype. At necropsy, major organs appeared normal, with no gross morphological defects after Cep55 deletion (Figure S7F). These results indicate that Cep55 is dispensable for adult tissue homeostasis, consistent with its low baseline expression in most adult tissues except testis. This supports the notion that anti‐tumor effects of Cep55 loss are tumor‐specific and highlights CEP55 as a promising therapeutic target with minimal expected toxicity in normal adult tissues.

### 3.9 Genetic Ablation of Cep55 Prolongs Survival in a Pten‐Deficient Tumor‐Prone Mouse Model

Previous studies have shown that CEP55 promotes PI3K stabilization and enhances AKT activation [11, 12], functionally mimicking the pathway activation caused by PTEN loss. In this context, CEP55 may become particularly critical to maintain hyperactivated PI3K/AKT signaling and promote malignancies. We therefore hypothesized that genetic ablation of *Cep55* in a *Pten‐KD* background could suppress tumor progression by blunting PI3K/AKT, offering an alternative strategy where direct inhibitors of this pathway have shown limited clinical success.

To test this, we generated a conditional, tamoxifen‐inducible mouse model allowing the co‐deletion of Cep55 and Pten in adult tissues, enabling us to examine whether inducible *Cep55* KO could reduce tumor incidence or delay onset in a PTEN‐deficient tumor prone model (Figure 4A). We crossed our Cep55^Fl/Fl^ line with Pten^Fl/Fl^ mice to allow simultaneous deletion of both genes. Because Cep55 (locus 19C2) and Pten (locus 19C1) reside on the same chromosome, we calculated a ∼3% likelihood of recombination to obtain the floxed alleles in cis. Out of ∼200 pups genotyped, we identified one with a successfully recombined Cep55‐Pten^Fl/+^ allele, which was subsequently bred with RosaCreERT2 mice. This breeding strategy produced RosaCreERT2*^+/–^* Cep55 *^Fl/Fl^*‐Pten^Fl/Fl^ offspring (hereafter referred to as Cep55‐Pten: CP‐DKD) and *Pten^Fl/Fl^*‐*RosaCre^ERT2+/–^* (Pten KD). Following tamoxifen administration at 6 weeks of age, we confirmed efficient deletion of both genes by immunoblotting across some tissues, with Cep55 detectable expression including spleen, liver, and testis (Figure S8A).

**FIGURE 4:**
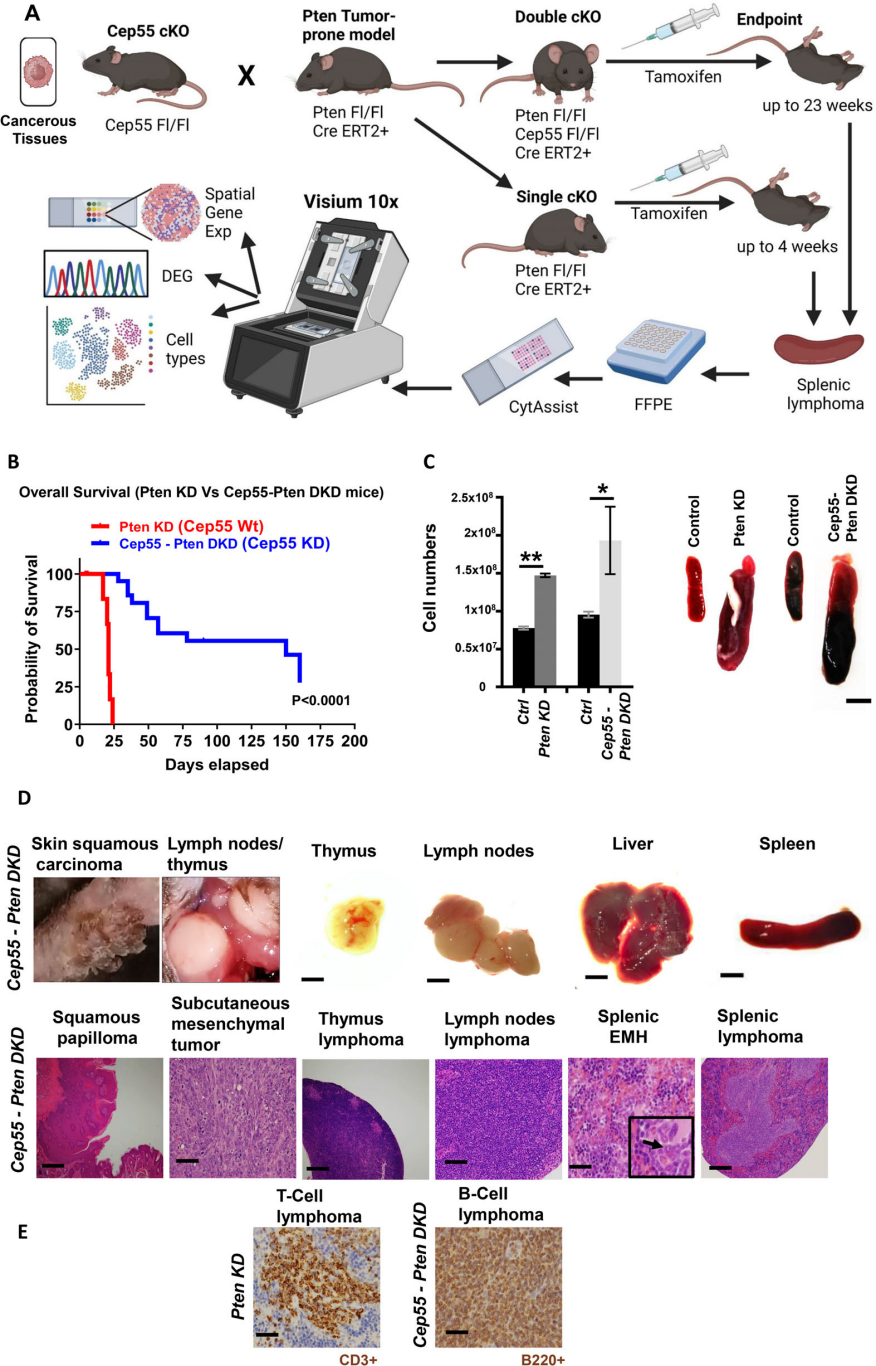
Cep55 loss extends survival and remodels tumor spectrum in a Pten‐deficient background. (A) A Schematic representation of the generation of conditional Pten knockout (Pten KD) and Cep55/Pten double knockout (DKD) mouse models using tamoxifen‐inducible Cre‐ERT2. Spatial transcriptomics and histopathological analyses were performed on tissues collected at defined time points (up to 23 weeks for DKD and 4 weeks for PTEN‐KD). (B) Kaplan–Meier survival analysis of Pten‐KD mice (n = 8) and Cep55–Pten double‐knockdown (CP‐DKD) mice (n = 16). Survival curves were compared using the log‐rank (Mantel–Cox) test. Median survival and corresponding P values are indicated in the figure. Animals were monitored until reaching predefined ethical endpoints or study completion. (C) Representative spleens from age‐matched control, Pten KD, Cep55 KD, and Cep55/Pten DKD mice showing severe splenomegaly observed in Pten KD, with partial rescue in DKD animals. Bar graph quantifies total splenic cell numbers. Scale bar = 5 mm. Data represent mean ± SD; n = 6 mice per group. (D) Macroscopic and microscopic analysis of tumor phenotypes in Pten KD versus Cep55/Pten DKD mice. Pten KD mice predominantly develop skin squamous carcinoma and thymic/lymphoid tumors, while DKD mice show subcutaneous mesenchymal tumors, thymic and lymph‐node lymphomas, splenic extramedullary hematopoiesis (EMH), and splenic lymphoma. Scale bars (upper panels) = 3 mm; H&E‐stained sections: squamous papilloma (180 µm), subcutaneous carcinoma and spleen EMH (60 µm), thymic and lymph‐node lymphoma (120 µm), and splenic lymphoma (180 µm). (E) Immunohistochemical staining confirming T‐cell lymphoma (CD3^+^) in Pten KD mice and B‐cell lymphoma (B220^+^) in Cep55/Pten DKD mice.

Consistent with previous reports, tamoxifen‐induced (Cre ERT2 recombination) Pten‐KD mice developed aggressive lymphomas and exhibited a median survival of only 21 days [15, 41]. In contrast, Cep55‐Pten DKD mice showed strikingly prolonged survival, with a median of ∼150 days, a greater than sevenfold extension (*p* < 0.0001, log‐rank test; Figure 4B). While all Pten KD mice died within 4 weeks post‐induction, approximately 20% of CP‐DKD mice remained alive and lymphoma‐free at the 160‐day experimental endpoint. This dramatic survival benefit indicates that loss of Cep55 greatly delays and hampers the tumorigenesis driven by complete Pten loss.

Pathological examination of moribund mice confirmed extensive splenomegaly and T‐cell lymphomas in Pten KD animals (Figure 4C). In contrast, CP‐DKD mice displayed more heterogeneous and delayed‐onset pathologies. While thymic and splenic lymphomas were still observed in some cases, many CP‐DKD mice developed benign or low‐grade lesions in other tissues at later time points, including uterine endometrial neoplasms (females), skin papillomas, (by 4–5 months) and rare cases of prostatic hyperplasia (males) (Figure 4D,E and Table S5). Notably, female CP DKD mice exhibited earlier mortality, with 100% dying by 7 weeks (often with T‐cell lymphomas), whereas 60% of male CP DKD mice survived beyond 5 months, suggesting sex‐specific influences on tumor development. These slower, often benign lesions suggest that in the absence of Cep55, the rapid lethal lymphomagenesis driven by Pten loss is mitigated, unmasking later‐onset pathologies in other organs. Surviving CP DKD mice (especially males) displayed unusual phenotypes mentioned above, including skin and prostate lesions, hinting that CEP55 may also impact specific tissue contexts differently (Figure 4D,E and Table S5). Collectively, the data suggest *Cep55* can delay tumorigenesis and increase tumor latency on a *Pten*‐deficient background.

At a molecular level, Cep55 loss dampened oncogenic signaling in Pten‐KD tumors. Immunohistochemistry (IHC) of spleen and liver tissues showed that CP‐DKD tumors had significantly reduced levels of phosphorylated AKT (Ser^473^), phospho‐ERK, and β‐catenin compared to Pten KD tumors (Figure S8B,C). FAK expression was also reduced in a subset of CP‐DKD tumors (Figure S8D), supporting the role of CEP55 in amplifying PI3K/AKT, MAPK, and focal adhesion signaling in vivo.

Together, these findings demonstrate that CEP55 is a critical enhancer of PI3K/AKT‐driven tumorigenesis in the context of PTEN loss. Its deletion significantly prolongs survival and alters tumor progression, positioning CEP55 as a viable therapeutic target in PI3K pathway–driven malignancies.

### 3.10 Spatial Transcriptomics Reveals Immunoregulatory and Differentiation Programs in CP‐DKD Tumors

To further investigate the molecular basis of delayed tumorigenesis in CP‐DKD mice, we performed spatial transcriptomic profiling using CytAssist2 and Visium platforms following the described workflow (Figure 4A). Data were normalized using nCount and nFeature metrics to control for sequencing depth and gene complexity, with Hb% included to account for hemoglobin transcript abundance, minimizing bias from highly expressed erythrocyte‐derived genes (Figure S9A). Compared to PTEN‐KD tumors, CP‐DKD tumors exhibited significant upregulation of genes (Average log_2_ fold change > 2.5) associated with immune/complement‐regulatory genes (e.g., Cd55, Igha, Dapl1) and downregulation (Average log2 fold change −2.5) of cell cycle, cytokinesis, and ECM‐related genes (e.g., Col1a1, Col1a2, Clec9a, Ptpn1) (Figure 5A–C). Notably, Aida (JUN/JNK signaling), Cd55 (immune evasion), and Dapl1 (epithelial differentiation) were among the most upregulated genes. Downregulated genes included collagen family members, CD207 (Langerin, a dendritic/Langerhans‐cell‐associated marker), and SERPINE1 (glioma progression), while cancer/testis antigens EIF2S3Y, DDX3Y, and KDM5D were also differentially expressed (Figure 5A–C and Figure S11A, Sup material_4).

**FIGURE 5:**
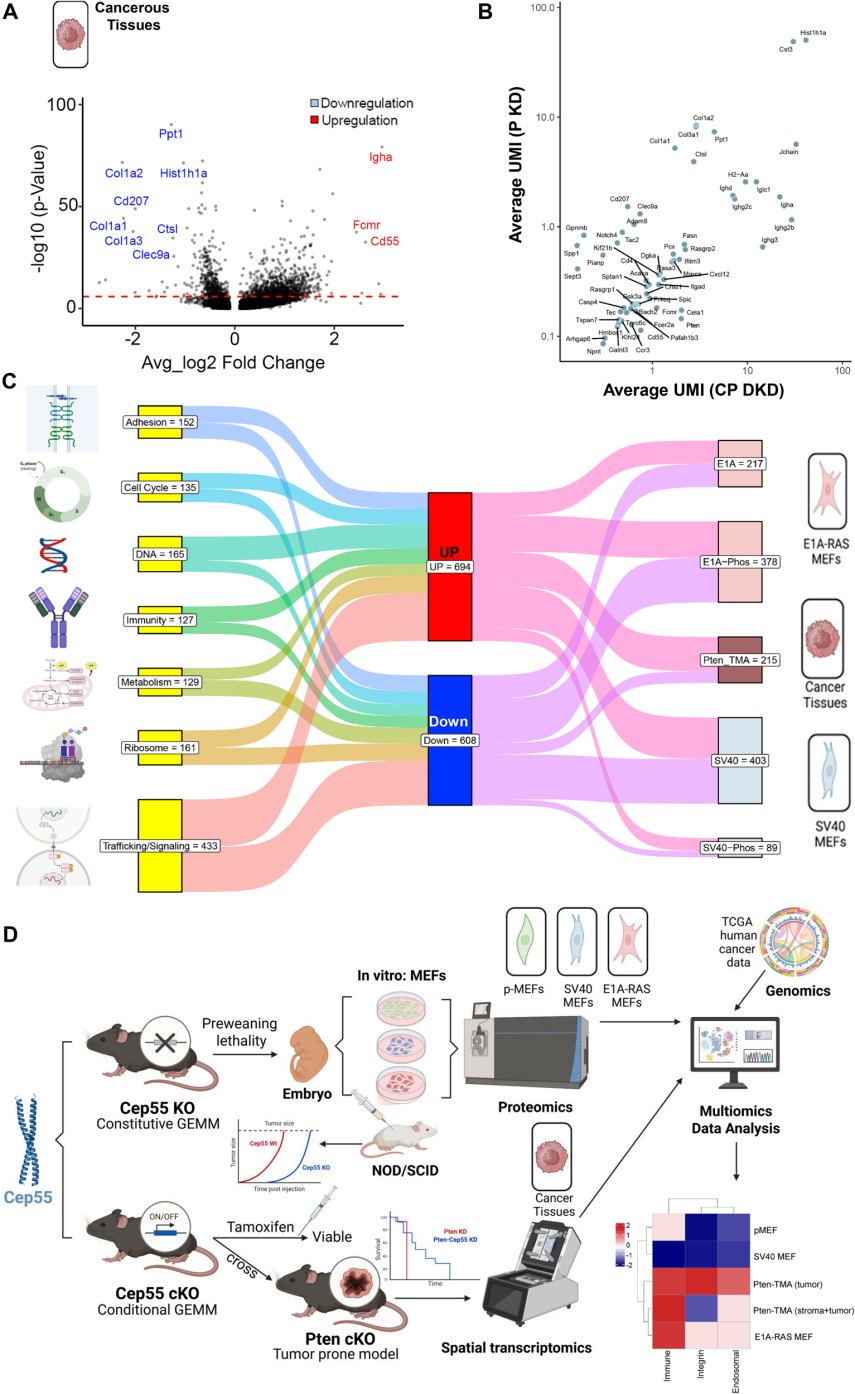
Integrated omics and spatial transcriptomics reveal Cep55‐dependent reprogramming of integrin‐mediated adhesion and T‐cell differentiation in Pten‐deficient tumors. (A) Volcano plot showing differentially expressed genes (DEGs) between Pten knockout (Pten KD) and Cep55/Pten double knockout (DKD) tumors (log_2_ fold change, Pten KD / Cep55‐Pten DKD). Downregulated genes (blue) include key adhesion and extracellular matrix components (Col1a1, Col6a2, Cdh1), while upregulated genes (red) are enriched for immune‐related and B‐cell/T‐cell activation markers (Igha, Cd55, Fcer1g), highlighting Cep55's role in integrin signaling and immune regulation. (B) Scatter plot highlighting the top 60 DEGs ranked by average UMI (unique molecular identifier) counts, demonstrating transcriptional heterogeneity between Pten KD and Cep55/Pten DKD tumor regions. Genes associated with integrin trafficking and immune cell communication display distinct expression patterns. (C) Sankey plot integrating multi‐omic datasets from Cep55 knockout MEFs (SV40 and E1A/Ras), spatial transcriptomics of Pten KD/Cep55_Pten (CP) DKD tumors, and TCGA human cancer datasets. Conserved Cep55‐regulated pathways, including adhesion, cell cycle, DNA repair, immune signaling, metabolism, and trafficking, are visualized across model systems, identifying Cep55 as a central coordinator of oncogenic signaling and immune‐ modulatory network. (D) Schematic overview of the experimental workflow used to investigate CEP55‐associated cancer molecular programs. Constitutive Cep55 knockout (KO) GEMMs showed preweaning lethality, prompting analysis of embryo‐derived MEFs, whereas tamoxifen‐inducible Cep55 conditional knockout (cKO) mice enable postnatal deletion and crossing with Pten‐driven tumor‐prone models. Primary, SV40‐immortalized, and E1A‐Ras–transformed MEFs were profiled by proteomics and phosphoproteomics. Tumor tissues from the models were analyzed by spatial transcriptomics and integrated with TCGA human cancer datasets. Multiomics correlation analysis revealed coordinated alterations of immune, integrin/adhesion, and endosomal expression signatures across model systems.

Within the annotated splenic lymphoma regions, *Cep55* transcript levels were significantly reduced in Cep55‐Pten DKD tumors compared to Pten‐KD tumors, with partial re‐expression observed in some tumors (Figure S9B). These transcript‐level findings were validated by IHC for CEP55 protein (Figure S9C). Pten transcript was low in Cep55‐Pten DKD tumors, but protein levels were undetectable or nonspecific (Figure S9D,E). B‐cells enrichment in CP‐DKD tumors was evidenced by upregulation of protein tyrosine phosphatase receptor type C gene (Ptprc) and confirmed by increased B220 protein levels on IHC (Figure S9F). Ptprc (CD45), is a transmembrane phosphatase expressed across hematopoietic cells, including B cells, T cells, and other leukocytes, is critical for immune signaling [42]. Interestingly, the B220 isoform of Ptprc is commonly used to identify B‐cells in mice, linking Ptprc directly to B‐cell profiling in both transcriptomic and protein‐level analyses.

Pathway analysis revealed upregulation of CD4+ T‐cell differentiation, integrin‐mediated cell adhesion, myeloid cell development, platelet activation, TORC1 signaling, and the p38 MAPK cascade in PTEN‐KD tumors relative to CP‐DKD tumors. In contrast, pathways related to cytokinesis, microtubule cytoskeleton organization, and cell cycle checkpoint signaling were downregulated (Figure S10A–C). Corresponding changes in molecular functions, including those involving integrin, fibronectin and growth factor binding, Rac1, RhoA, Fgfr1 and death receptor signaling, were also observed (Figure S10A–E).

Comparison of unique differentially expressed genes in stroma and tumor regions highlighted changes in tumorigenesis‐related genes, such as downregulation of MMP2, MMP12, Spp1, and upregulation of Gpd1, Cdo1, and Il7r (Figure S10). The dual roles of these genes in tumor progression underscore the importance of evaluating transcriptomic signatures within cellular context.

Pathways disrupted in CP‐DKD tumors, including integrin‐mediated adhesion, cytokinesis, and cell–substrate interactions, overlapped with those identified in proteomic analysis of Cep55‐deficient E1A/Ras MEFs (Table S6). Similar pathway level patterns were observed in TCGA datasets, confirming consistency across mice and human cancer studies (Figure 5D and Sup material_ 5).

Panel S11b integrates pathway signatures of Endosomal/Vesicle Trafficking, Immune Modulation, and Integrin/Adhesion modules from the Cep55‐Pten transcriptomics and MEF proteomics. The heatmap shows positive enrichment for immune‐regulatory and leukocyte‐associated pathways, and negative for collagen, extracellular‐matrix, adhesion, endocytosis, and migration‐related pathways. These patterns are consistent with coordinated pathway‐level remodeling across multiple biological categories, rather than isolated single‐gene changes.

Focusing on cancer immunity, we analyzed immune‐module scores across tumor and tumor+stroma compartments from Pten‐KD and CP‐DKD transcriptomics samples (Figure S11C). While multiple adaptive and innate immune modules were generally downregulated in CP‐DKD in both tissue contexts in comparison to Pten‐KD, the effective anti‐tumor immunity cell CD8+ cytotoxic, cytokine Th1 and pro‐inflammatory M1 macrophage signatures were upregulated in tumors. These data show that Cep55‐loss in a Pten‐deficient background results in broad remodeling of the immune landscape.

Biologically meaningful effect‐size scores confirmed CP‐DKD favoring specifically anti‐tumor immunity signatures over Pten‐KD, such as Chemokine Myeloid/T cell, Cytokine Th1, CD8+ cytotoxic, and Macrophage M1 modules (Figure S11D). Accordingly, Pten‐KD showed immune escape and inflammatory signatures such as T‐cell exhaustion, Macrophage M2, type I IFN and suppressive cytokine (Figure S11C). Thus, Cep55‐loss in the Pten‐KD tumor model background selectively increases beneficial anti‐tumor immunity signatures while also downregulating several other immune modules. Confirming the importance of tumor microenvironment in immunity, inflammatory and adaptive immune signatures for T‐cell activation, Leukocyte regulation, Complement, B‐cell humoral, and Inflammatory were upregulated in Pten‐KD tumor‐tissue models rather than the E1A/RAS MEF 2D oncogenic cell model (Figure S11E).

Collectively, the spatial transcriptomic analyses show that Cep55 loss in a Pten‐deficient background is associated with altered immune‐associated gene expression, enrichment of differentiation programs, and reduced expression of cell cycle and ECM‐related pathways.

### 3.11 Cep55 Knockdown Is Associated With Altered Macrophage Polarization and Context‐Dependent Antigen‐Specific T‐Cell Responses In Vitro

To determine whether tumor‐cell CEP55 expression influences macrophage education and/or cytotoxic T‐cell stimulation, we used the AT3‐OVA system. This model provides an immunogenic murine mammary tumor context as AT3 cells express ovalbumin, allowing tumor–immune interactions to be assessed in an antigen‐relevant setting [43]. In the AT3‐OVA/OT‐I model, the SIINFEKL peptide is presented on MHC class I to OT‐I CD8 T cells, making this system widely used for studying antigen‐specific T‐cell activation and tumor–immune recognition (Figure S11F).

We generated two independent doxycycline‐inducible Cep55 knockdown polyclonal populations, C7 and C8, using SMARTvector Tet‐inducible shRNAs targeting Cep55 (Figure S11G). Conditioned media were collected from AT3‐OVA C7 and C8 cells cultured with or without doxycycline. These conditioned media were then applied to mouse bone marrow–derived macrophages, followed by qRT‐PCR analysis of macrophage polarization‐associated transcripts (Figure S12A). Western blotting confirmed successful Cep55 knockdown in doxycycline‐treated C7 and C8 cells (Figure S12B). In macrophages exposed to conditioned medium from C7 +Dox cells, corresponding to Cep55‐KD conditions, tumor‐supportive/M2‐like associated markers, including *Retnla* and *Mrc1/Cd206*, showed a downward trend, whereas inflammatory/M1‐associated markers, including *Cxcl10, Tnf* and *IL1b*, showed modest upward shifts. Consistently, macrophages exposed to conditioned medium from C8 +Dox cells showed reduced expression of the immunosuppressive markers *Mrc1/Cd206* and Retnla, together with increased *Il1b*, *Cxcl10* expression and modest changes in *Tnf*. These data show that tumor cells with Cep55 knockdown shift macrophage polarization away from an immunosuppressive/ tumor‐supportive phenotype.

We next examined antigen specific T‐cell responses using co‐cultures of AT3‐OVA (C7 and C8‐Cep55 shRNA transduced) mammary tumor cells, (with or without doxycycline induction), and splenic OT‐I CD8 T cells in the presence of SIINFEKL peptide to promote antigen‐specific activation. Doxycycline reduced CEP55 protein levels by approximately 74% in C7 cells and 96% in C8 cells (Figure S12B). In the T‐cell killing assay, C7 +Dox cells showed a trend toward reduced tumor‐cell abundance, whereas C8 +Dox cells (with near complete loss of Cep55) showed a significant reduction in tumor‐cell abundance at an effector‐to‐target (E:T) ratio of 3:1 (Figure S12C). This was associated with a trend toward increase in CD8 T cell and effector memory T cells (CD44+CD62L‐) abundance and TNF alpha expression by CD8 T cells (Figure S12C–E). Together, these findings suggest that loss of CEP55 modulates both innate and adaptive immune components, shifting the tumor microenvironment toward a less immunosuppressive and more immune‐reactive state.

To extend these observations to human‐relevant systems, we performed validation studies in human B‐cell lymphoma cell line such as Raji, Ramos, Daudi, BJAB (Figure S12F–I). For CEP55 knockdown, we utilized adenoviral GFP‐linked shRNA constructs targeting CEP55 or a scrambled control shRNA, as previously described [9]. GFP positivity indicated successful construct delivery and/or expression across the cell‐line panel and CEP55 knockdown was confirmed at the protein level in BJAB, Raji, and Daudi cells, and Ramos cells showed minimal knockdown. CEP55 knockdown reduced cell viability in a subset of lymphoma cell lines, with the strongest sensitivity observed in PTEN‐deficient BJAB cells. We further examined this model and found that CEP55 knockdown using siRNA's modestly increased MHC‐I expression while reducing viability of cells with greater knockdown efficiency (SiRNA#1) (Figure S12H,I). These findings suggest Cep55 knockdown had a greater impact on viability of PTEN‐deficient BJAB cells compared with other lymphoma cell lines.

In summary, Cep55 knockdown in vitro was associated with changes in macrophage polarization markers and enhanced antigen‐specific CD8^+^ T‐cell responses. In lymphoma cell lines, CEP55 depletion reduced cell viability, with a more pronounced effect observed in PTEN‐deficient BJAB cells and was accompanied by modest increases in MHC‐I expression.

Taken together, these data show that CEP55 loss is associated with alterations in both tumor‐intrinsic viability and immune‐related phenotypes across in vitro and in vivo models of PTEN deficiency.

### 3.12 Discussion

CEP55 overexpression has been implicated in tumorigenesis across a broad range of cancers, including T‐cell lymphoma, breast, lung, colorectal, and liver cancers. Traditionally recognized as a cytokinesis regulator in cancer cells; CEP55 emerges as a multifunctional oncogenic driver with expanding roles in tumorigenesis [11]. It promotes malignant transformation through activation of pro‐survival pathways such as PI3K/AKT and by contributing to genomic instability.

Here, we provide comprehensive in vitro and in vivo evidence that, while Cep55 is essential for embryonic development, it is largely dispensable for normal adult tissue homeostasis. Using an inducible Cep55 cKO mouse model, we circumvented the limitations of perinatal lethality observed in constitutive knockouts and showed that its loss in adult mice result in no overt physiological abnormalities [9, 44, 45]. This is consistent with its restricted expression profile, with high levels in testis and only moderate expression in the adult spleen and intestine, supporting a limited role in normal adult physiology.

Proteomic profiling of Cep55‐deficient MEFs revealed transformation stage‐specific alterations in key cellular pathways. In primary and early transformed MEFs, Cep55 loss led to downregulated ribosome biogenesis and RNA processing pathways, implicating a role in translational control and cellular metabolism. These effects likely reflect CEP55‐dependent functions at centrosomal and midbody function, which spatially organize RNA and protein complexes involved in cell division and growth [28, 29, 30].

Notably, Cep55 loss induced distinct, transformation‐dependent effects on adhesion and signaling pathways. In early‐transformed cells, ECM remodeling, integrin signaling, and actin cytoskeleton organization were upregulated, whereas in fully transformed, tumorigenic MEFs, these pathways were suppressed alongside enrichment of chromatin remodeling and DNA damage response pathways. These findings suggest dynamic rewiring of adhesion and epigenetic regulation during tumor progression. Mechanistically, Cep55 loss impaired integrin internalization, prolonged retention near the plasma membrane, and disrupted trafficking to early and late endosomes, paralleling delayed endocytosis of other cargos such as dextran. More broadly, Cep55 deficiency induced global remodeling of endosomal system, reflected by coordinated alterations in ESCRT components, lipid metabolism and cell signaling pathways. Canonical ESCRT‐dependent membrane processes, including viral budding, reportedly remain functional following CEP55 loss, being distinct from compromised cytokinetic ESCRT‐mediated activities [4, 46, 47]. These findings suggest that CEP55 regulates integrin trafficking indirectly through broader modulation of endosomal system such as expression changes in ESCRTs and other adaptor proteins. Notably, altered ESCRT functions have been linked to tumor immune evasion via PD‐L1 regulation and modulation of CD8^+^ T‐cell infiltration [48].

Integrin signaling is closely linked to centrosome function [49], and mitotic progression [50], processes that may be compromised in the absence of CEP55. Supporting this notion, Kamranvar et al. [51] reported that integrin‐mediated adhesion activates the FAK–Src signaling axis, which engages PI3K–AKT and contributes to the regulation of cell‐cycle kinases, including PLK1. PLK1, in turn, directly phosphorylates CEP55 during mitosis to control its temporal recruitment and function during cytokinesis. In this framework, adhesion‐dependent signaling acts upstream of CEP55 via FAK–Src–PLK1. Our findings extend this model by demonstrating a reciprocal relationship, whereby CEP55 feeds back to regulate integrin trafficking and signaling competence. Loss of CEP55 impairs integrin α5β1 endocytosis, disrupts endosomal organization, and attenuates downstream FAK, AKT, and ERK signaling in transformed MEFs. Thus, CEP55 functions both as a downstream effector and an upstream regulator of integrin signaling, forming a self‐reinforcing regulatory loop linking adhesion signaling, membrane trafficking, and cell‐cycle control. This bidirectional interaction may give rise to a form of “malignant synergy” whereby CEP55‐driven proliferative capacity and integrin‐dependent invasive signaling are co‐amplified across successive cell cycles. Such a mechanism would enable tumor cells to simultaneously sustain high mitotic activity while enhancing migration, adhesion remodeling, and metastatic potential.

Consistent with this, reduced Cep55 levels in transformed and immortalized MEFs translated into reduced cell migration, adhesion, invasion, and clonogenic growth in both 2D and 3D culture assays. Corroborating the in vitro findings, Cep55‐KO MEFs (E1A/Ras transformed) formed tumors with delayed onset, slower growth, and increased necrosis, suggesting compromised tumor fitness. Notably, the mitotic index remained unchanged at end point, suggesting that reduced tumor progression is not primarily driven by altered proliferation, but rather by defects in survival, adhesion, and microenvironmental interactions. Importantly, the tumor studies were conducted in NOD/SCID mice, which lack functional adaptive immunity (T and B cells) but retain components of the innate immune system, including neutrophils, NK cells, and immature macrophages and dendritic cells. Although these innate populations are functionally compromised, their presence raises the possibility that innate immune mechanisms may contribute, at least in part, to the observed tumor phenotype. Collectively, these findings highlight a key role for CEP55 in supporting integrin‐mediated processes critical for tumor progression. Consistent with this, bioinformatic analyses revealed co‐occurrence and positive correlation between CEP55 copy number gains and integrin signaling components in human breast cancer datasets, supporting a conserved role for CEP55 in integrin‐dependent oncogenic pathways.

A key discovery in our study was the genetic interaction between Cep55 and the well‐established tumor suppressor PTEN. While PTEN loss alone led to increased Akt signaling and promoted rapid development of tumorigenesis, simultaneous Cep55‐deletion mitigated this phenotype. In the Cep55‐PTEN double knockout (DKD), we observed delayed lymphoma onset, reduced growth, and reduced downstream PI3K/Akt signaling compared to PTEN‐KD alone. These findings suggest that Cep55 amplifies PI3K signaling and functions either downstream or parallel with PTEN, to contribute to lymphoma development and progression. The predominance of lymphoma in our in vivo models reflects the well‐established tumor spectrum of systemic Pten‐deficient mice, rather than a lymphoma‐restricted role for CEP55.

We also observed sex‐specific differences in tumor spectrum and survival following *Cep55* deletion. Female DKD mice exhibited earlier mortality, often due to T‐cell lymphomas, whereas 60% of males survived beyond 5 months, suggesting sex‐specific influences on tumor biology that warrant further investigation. Such dimorphism is well documented in cancer and may reflect context‐dependent effects of sex hormones, immune responses, and sex chromosome–linked gene regulation. Estrogen and androgen signaling can modulate integrin/FAK and PI3K/AKT pathways, which are functionally linked to CEP55, and may therefore differentially influence tumor progression.

Moreover, spatial transcriptomics of tumors revealed that Cep55 deletion in the PTEN‐null background induced immune‐modulatory transcriptional programs, with upregulation of immune regulators (e.g., Cd55, Dapl1) and downregulation of immunosuppressive genes (e.g., Serpine1, Cd207). Enhanced expression of Ptprc (CD45) and B220 also indicated increased B‐cell infiltration, suggesting a role in the regulation of the tumor microenvironment. However, whether these cells directly contribute to tumor suppression or instead represent a broader shift toward an immune‐permissive microenvironment remains to be determined. Functionally, we show that Cep55 suppression shifts macrophage polarization away from an M2‐like, tumor‐supportive phenotype while modestly enhancing inflammatory signaling, and is associated with increased tumor cell killing in antigen‐specific co‐culture systems. Together, these results suggest that tumor‐intrinsic CEP55 contributes to immune evasion by coordinating both innate and adaptive immune suppression.

These observations are consistent with emerging literature implicating CEP55 in immune regulation. For example, a USP8–CEP55–CHMP6 axis has been shown to promote triple‐negative breast cancer progression by enhancing macrophage M2 polarization and suppressing ferroptosis [52]. Similarly, CRISPR–Cas9‐mediated CEP55 knockout in liver cancer models reduced PD‐L1 expression, increased MHC‐I levels, decreased ROS and immunosuppressive cytokines, and enhanced T‐cell effector function and cytotoxicity [53]. In addition, genome‐wide CRISPR screening studies have demonstrated that loss of CEP55 increases tumor sensitivity to cytotoxic T‐lymphocyte (CTL)–mediated killing, further supporting a role for CEP55 in promoting immune resistance [54]. Future in vivo studies will be required to determine whether targeting CEP55 can meaningfully enhance anti‐tumor immunity or improve responses to immunotherapy.

A limitation of our study was the rapid lymphoma onset and lethality in the PTEN‐KD model, which restricted long‐term mechanistic studies. As lymphoma represents the predominant and most lethal malignancy in this model, we focused on lymphoid organs for detailed analysis. Despite this constraint, the striking sevenfold survival extension in DKD mice enabled identification of altered tumor spectrum and grade with later‐onset, less aggressive tumors (e.g., papillomas, hemangioendotheliomas, prostate hyperplasia). Complementary TCGA analyses demonstrate that CEP55 is broadly overexpressed in human solid tumors and associated with poor clinical outcomes. Importantly, emerging clinical and experimental data support a role for CEP55 in lymphoid malignancies, where its expression correlates with proliferative indices (e.g., Ki‐67) and functional studies demonstrate that CEP55 depletion reduces tumor cell viability and promotes apoptosis [55]. This suggests that CEP55 contributes to tumor cell fitness in lymphoma, consistent with the phenotypes observed in our model.

We therefore interpret our findings as identifying a core CEP55‐driven mechanistic axis (integrin–endosomal–PI3K/AKT) that is conserved across tumor types (lymphomas and solid tumors), while the specific tumor spectrum observed in vivo is shaped by the genetic context of Pten loss. Although, a broader analysis of tissue‐specific tumor incidence would require conditional knockouts across organ systems, these findings reinforce the cancer‐specific importance of CEP55, particularly in PTEN‐deficient contexts.

Importantly, PTEN‐deficient tumors can engage adhesion‐linked compensatory survival pathways outside the canonical PI3K–AKT axis. In PTEN‐null T‐cell acute lymphoblastic leukemia, FAK was shown to provide a parallel survival signal, and genetic or pharmacologic inhibition of FAK sensitized PTEN‐null leukemic cells to PI3K/AKT/mTOR inhibition, supporting the relevance of integrin/adhesion‐associated signaling as a therapeutic vulnerability in PTEN‐deficient contexts [56]. These studies support our model that CEP55 promotes PTEN‐deficient tumor progression not only through tumor‐intrinsic proliferative programs, but also through integrin/adhesion‐linked survival, endosomal trafficking, ferroptosis resistance, and immune‐suppressive tumor microenvironmental remodeling.

Together, these findings position CEP55 as a context‐dependent modulator of integrin trafficking and cell adhesion, whereby global remodeling of the endosomal system may secondarily influence ESCRT‐dependent sorting without Cep55 acting as a core component of ESCRT machinery. Its dispensability in adult tissues and its synthetic vulnerability in PTEN‐deficient tumors highlight it as a compelling therapeutic target. Future studies should explore CEP55 inhibition in preclinical models, assess combinatorial strategies with immunotherapy or PI3K inhibitors, and refine our understanding of the CEP55–integrin–PI3K axis in diverse cancer settings.

## 4 Conclusion

This study identifies CEP55 as a promising, cancer‐specific therapeutic target, particularly in PTEN‐deficient tumors. Through integrated proteomic, genetic, and spatial transcriptomic analyses, we show that CEP55 deletion impairs PI3K–AKT signaling, disrupts integrin trafficking, and enhances anti‐tumor immunity. Importantly, normal adult tissues tolerate CEP55 loss, suggesting a potentially favorable therapeutic window that requires pharmacologic validation. By prolonging survival and reducing tumor burden in a PTEN‐null mouse model, CEP55 emerges as a multifunctional driver of malignancy and a potential vulnerability in aggressive cancers. Targeting CEP55 could provide a novel strategy to suppress tumor progression, particularly in PI3K/AKT‐driven and immune‐evasive cancers that are resistant to existing therapies.

## 5 Extended Materials and Methods

### 5.1 Animal Husbandry and Ethics Statement

All mouse experiments adhered to the Australian Code for the Care and Use of Animals for Scientific Purposes and were approved by the QIMR Berghofer Animal Ethics Committee (protocol A0707‐606 M). All efforts were made to minimize animal suffering and the number of animals used.

#### 5.1.1 Housing Conditions

Mice were housed in OptiMICE individually ventilated cages (Centennial, Colorado, USA) in a pathogen‐free facility (22–25°C, 40%–60% humidity, 12 h light/dark cycle) with ad libitum access to food and water. The following strains were used: C57BL/6J (RRID:IMSR_JAX:000664), Rosa26‐CreERT2 (RRID:IMSR_JAX:008463), NOD/SCID (RRID:IMSR_JAX:001303), CEP55 transgenic and floxed lines generated as previously described [9].

### 5.2 Generation of Conditional Cep55 Knockout Mice

Cep55 floxed embryonic stem cells, obtained from the International Knockout Mouse Consortium, were used by the Australian Phenomics Network to produce heterozygous Cep55‐targeted mice. These transgenic (Tg) mice possessed a “knockout‐first” allele that facilitated constitutive Cep55 knockout or conditional allele generation upon further crossing. Conditional Cep55 knockout mice (Cep55 cKO) were generated by crossing heterozygous Cep55^Tg/+^ mice with FlpE mice to remove the neo cassette, resulting in Flp; Cep55^Fl/+^ progeny. Subsequent backcrossing with wild‐type C57BL/6 mice removed the Flp transgene. Further intercrossing of Cep55^Fl/+^ mice produced Cep55Fl/Fl offspring. These mice were crossed with RosaCre^ERT2^ transgenic mice, generating RosaCre^ERT2^; Cep55^Fl/+^ mice, which were bred again with Cep55^Fl/Fl^ mice to obtain RosaCre^ERT2^; Cep55^Fl/Fl^ progeny. Tamoxifen administration activated Cre recombinase, resulting in Cep55 conditional knockout mice.

#### 5.2.1 Sex and Demographics

Sex was incorporated as a biological variable according to ARRIVE (Animal Research: Reporting of in vivo experiments) guidelines. In GEMM experiments (Cep55‐cKO, Cep55–Pten DKD, and Pten controls), both male and female mice (6–8 weeks old) were used, with group assignments balanced by sex to reduce confounding effects. For the E1A/Ras MEF transplantation model, only female NOD/SCID mice (6–8 weeks old) were used due to known male–male aggression and wound‐related attrition in group‐housed males. Sex was recorded at the time of allocation and factored into randomization procedures. No sex‐specific exclusions occurred.

#### 5.2.2 Inclusion / Exclusion Criteria

Mice were included if they were successfully genotyped for the required alleles; Met health and weight (>18 g) criteria; Showed no signs of spontaneous disease.

#### 5.2.3 Exclusion Criteria

Unexpected health deterioration unrelated to the experimental intervention; Mistaken genotype; Injection failure (e.g., mis‐injection confirmed immediately by operator). All exclusions were documented; no animals were excluded post‐hoc based on outcome.

#### 5.2.4 Randomization and Blinding

Animals were randomly assigned using a random‐number generator to experimental vs. control groups. Investigators performing tumor measurements, histopathological scoring, and image quantification were blinded to genotype and treatment. Genotyping was performed by a separate individual not involved in downstream analysis.

#### 5.2.5 Power Calculation

Group sizes (typically n = 5–10 per group) were determined based on prior data and pilot experiments, aiming to detect ≥30% effect size with 80% power at α = 0.05. Formal power calculations were performed using G*Power (RRID:SCR_013726).

### 5.3 Genotype Analysis

Genomic DNA, extracted from mouse ear samples using QuickExtract DNA Extraction Solution (Lucigen, USA), was used for genotyping. PCR employed a 3‐primer strategy with a common forward primer (P1) and two distinct reverse primers (P2, P3). Wild‐type mice produced a single band (381 bp), heterozygous mice two bands (381 bp, 454 bp), and homozygous knockout mice one band (454 bp). Primer sequences for Cre, FLPe, Pten, and Kras were previously published, while new sequences are listed: Cep55 (TGGGTCTTTAACTCATGGTC, AGGAGTGAAAAGTCCTCACA, GTACCGCGTCGAGAAGTT); Cre recombined (AACTGATGGCGAGCTCAGA); FLPe (GTGGATCGATCCTACCCCTTGCG, GGTCCAACTGCAGCCCAAGCTTCC); Pten (GCCCCGATGCAATAAATA, ACTCAAGGCAGGGATGAG); rcm2_CreF (TGTGGACAGAGGAGCCATAAC, CATCACTCGTTGCATCGACC); ROSA26 locus (AGCACTGGAAATGTTACCAAGGAAC, GGCTGGCTAAACTCTGGCCCTACA)

### 5.4 Timed Mating

Successful timed mating was confirmed by observing copulation plugs the following morning, designated embryonic day 0.5 (E0.5).

### 5.5 Organ and Embryo Isolation

Mice were anesthetized with Isoflurane (Attane, Biomac) and euthanized via cervical dislocation. Organs and embryos, dissected using a Nikon SMZ45 stereo microscope, were rinsed in ice‐cold PBS. Samples were either snap‐frozen for protein/mRNA extraction or fixed in Bouin's solution or 4% paraformaldehyde (PFA) for histological analyses.

### 5.6 Mouse Cell Derived Transplant

Transplants were established in NOD/Scid mice (*RRID: IMSR_JAX:001303*) by subcutaneous injection of E1A/Ras‐immortalized MEFs (1 × 10^6^ cells suspended in Matrigel/PBS). Tumor growth was measured thrice weekly using calipers, and tumor volume calculated using the formula V = (W^2^ × L)/2.

### 5.7 Tamoxifen Induction

Cre recombination was induced in vivo using tamoxifen (1 mg) freshly prepared in sunflower oil/ethanol. MEFs underwent 4‐hydroxy tamoxifen (4‐OHT) treatment (1–3 µM) with optimal gene deletion observed at 1 µM after 72 h.

### 5.8 Histology, H&E and Immunohistochemistry Staining

Tissues were fixed, embedded in paraffin, sectioned (4 µm), and processed for H&E and periodic acid–Schiff staining as previously described [21]. Antigen retrieval utilized sodium citrate buffer. Sections were permeabilized, blocked, incubated overnight with primary antibodies, and detected using Alexa Fluor–conjugated secondary antibodies. Images were acquired using Aperio Scanscope and analyzed via Image Scope (Leica Biosystems) and Imaris software (*RRID: SCR_007370*).

### 5.9 Cell Culture and MEF Establishment

Cell lines from ATCC were maintained by guidelines, authenticated by STR profiling, and tested annually for Mycoplasma. MEFs were isolated from e13 embryos. The MEFs were cultured in DMEM (20% FBS, antibiotics, antifungals) and immortalized via SV40T retroviral or E1A/Ras transduction. A panel of human B‑cell lymphoma lines were obtained from the Rajiv Khanna laboratory (QIMR Berghofer Medical Research, Institute).

### 5.10 Cell Proliferation and Viability Assays

Proliferation was monitored using IncuCyte. Viability was assessed by MTS assay, measuring absorbance at 490 nm post‐treatment.

### 5.11 Clonogenic and Soft Agar Assays

MEFs seeded at low densities formed colonies after 14 days, quantified via crystal violet staining and imaging with GE InCell 2000 software.

### 5.12 3D Culture

MEFs cultured in Happy Cell ASM medium formed spheroids, assessed by Hoechst staining and Cytell imaging after 14 days.

### 5.13 Cell Invasion and Adhesion Assays

A custom invasion assay was established using a polyisocyanopeptide (PIC)‐based hydrogel. PIC polymer hydrogel was synthesized with the collagen‐mimicking peptide sequence Gly‐Phe‐Hyp‐Gly‐Glu‐Arg (GFOGER) using previously described methods. PIC with a molecular weight Mv = 476 kg/mol containing 1% GFOGER ligand density (PIC‐GFOGER) was dissolved in media to 2 mg/mL at 4°C. To generate spheroids, cells were seeded in ultra‐low attachment 96‐well plates (Corning Costar CLS7007) at a density of 1000 cells per well in complete DMEM medium. The cells formed spheroids of uniform size overnight and were maintained for 48 h. After 48 h, the spheroids were individually mixed with cooled PIC‐GFOGER polymer solution on ice. The mixture was gently transferred to µ‐Slide 15 Well 3D plates (Ibidi µ‐Slide 15 Well 3D) and incubated at 37°C to allow gelation. 30 min after initial gelation, complete medium was added on top of the gel in each well. Spheroid outgrowth was monitored using a Leica SP8 confocal microscope with a 10× objective under brightfield settings. Spheroid diameters were measured using ImageJ (*RRID: SCR_003070*). Adhesion assays involved collagen I‐coated plates, crystal violet staining, and absorbance measurement at 570 nm.

### 5.14 Live‐Cell Imaging and Immunofluorescence

Live‐cell imaging utilized EVOS Fl Auto and confocal microscopy. Immunofluorescence involved fixation, permeabilization, antibody staining, and imaging via DeltaVision microscopy.

### 5.15 Western Blotting

Tissues and cells were lysed in RIPA or urea buffers, protein quantified via BCA assay, electrophoresed via SDS‐PAGE, transferred to nitrocellulose membranes, and probed with antibodies listed: CEP55 CST (D1L4H) Rabbit mAb #81693 1:1000; Cep55 Santa Cruz biotechnology, sc‐374051, 1:500; Vinculin Cell Signaling Technology #13901, 1:2000; β‐Actin BD Pharmingen#612656, 1:2000; β‐Catenin Cell Signaling Technology 9582, 1:1000; pERK1/2 (T202/Y204) Cell Signaling Technology #4370, 1:1000; ERK1/2 Cell Signaling Technology #4695, 1:1000; pAKTS473 Cell Signaling Technology #4060, 1:1000; AKT Cell Signaling Technology #9271, 1:1000; MYC (Y69) Abcam #ab32072, 1:1000; Rabbit Secondary (peroxidase) Sigma Aldrich #A0545, 1:5000; Mouse Secondary (peroxidase) Sigma Aldrich #A9044, 1:5000, HLA class I ABC Monoclonal antibody (proteintech # 66013‐1‐Ig).

### 5.16 TMT‐Based Proteomic and Phosphoproteomic Analysis

Cells were lysed in 2% SDS/100 mM triethylammonium bicarbonate supplemented with protease and phosphatase inhibitors. Lysates were sonicated, clarified by centrifugation, and protein concentrations were determined by BCA assay. Equal amounts of protein were reduced with dithiothreitol, alkylated with iodoacetamide, acetone‐precipitated, trypsin‐digested, labeled with tandem mass tags, pooled, and fractionated by high‐pH reverse‐phase liquid chromatography. Ten percent of each sample was retained for total proteome analysis, while the remaining 90% was subjected to TiO_2_‐based phosphopeptide enrichment using a modified EasyPhos workflow. The localization probability cutoff was 0.75 (75%) and the enrichment efficiency was 97%.

Peptide fractions were analyzed by LC–MS/MS using an Orbitrap Fusion Tribrid mass spectrometer coupled to a nanoAcquity UHPLC system. Total proteome samples were acquired using MS1/CID‐MS2/HCD‐MS3 for TMT reporter‐ion quantification, whereas phosphoproteome samples were analyzed using HCD‐MS2 acquisition. Raw data were processed in Proteome Discoverer using SEQUEST HT against the UniProt database. Peptide‐spectrum matches were filtered at a 1% false discovery rate, phosphosite localization was accepted at phosphoRS ≥75%, and reporter‐ion intensities were normalized to total peptide abundance.

### 5.17 TCGA Analyses

Messenger RNA levels, that is, batch effect‐normalized log2(normalized counts+1) (Illumina HiSeq RNASeqV2), were obtained from the pan‐cancer dataset of The Cancer Genome Atlas (TCGA), including 32 cancer types [57], and processed as previously described [58].

### 5.18 Immune Cell Infiltration Levels in Cancers

Estimation of the levels of 26 tumor‐infiltrated immune cell types or states in TCGA samples occurred using CIBERSORTx, as previously described [59]. These levels were compared to expression levels of CEP55 in the same samples using Spearman correlations. Spearman R and p values were determined in R (The R Project for Statistical Computing).

### 5.19 Gene Set Enrichment Analyses

For TCGA samples, single‐sample Gene Set Enrichment Analysis (ssGSEA) was used as described [60], to determine ssGSEA scores that reflect the activity of specific biological pathways or gene sets on a per‐sample basis. The ssGSEA scores were compared to gene expression levels per sample. Heatmaps were created, with tiles showing the Spearman correlations (r and p values) between individual gene expression levels (CEP55 or PTEN) and the ssGSEA gene expression signatures per cancer type.

### 5.20 Generation of Doxycycline‐Inducible Cep55 Knockdown AT3‐OVA Cells

AT3‐OVA murine tumor cells, kindly provided by the Zhian Chen laboratory, were transduced with the SMARTvector Lentiviral shRNAs (Dharmacon, Horizon Discovery) Tet‐inducible lentiviral shRNA system to generate inducible Cep55 knockdown models. SMARTvector constructs carrying independent Cep55‐targeting shRNAs, including C7 and C8, were expanded in DH5α competent cells and packaged in HEK293T cells for lentiviral particle production. AT3‐OVA cells were transduced with harvested lentiviral particles and selected with puromycin to establish stable AT3‐OVA‐C7 and AT3‐OVA‐C8 lines. For knockdown induction, cells were treated with doxycycline for 72 h, while matched untreated cultures were maintained as controls. Cep55 depletion was confirmed by immunoblotting before use in downstream cancer immunity assays.

### 5.21 AT3‐OVA/OT‐I T‐Cell Co‐Culture Killing Assay

AT3‐OVA cells and doxycycline‐inducible Cep55 knockdown derivatives, including AT3‐OVA‐C7 and AT3‐OVA‐C8, were used to assess tumor‐cell susceptibility to OT‐I–mediated immune killing. AT3‐OVA cells were seeded in 24‐well plates at 4 × 10^4^ cells per well and treated with doxycycline where indicated. The following day, spleens from OT‐I mice were harvested, mechanically dissociated into single‐cell suspensions, subjected to red blood cell lysis, washed, and resuspended in complete RPMI medium. OT‐I splenocytes were added to AT3‐OVA cultures at a splenocyte ratio of 30:1, corresponding approximately to a CD8^+^ T cell ratio of 3:1. OVA_257_–_264_ peptide was added to a final concentration of 0.02 nM, and co‐cultures were maintained in 1 mL complete RPMI at 37°C with 5% CO_2_ for 72 h. Where indicated, tumor cells were labeled with CellTrace Violet before co‐culture to enable discrimination of tumor cells by flow cytometry. At endpoint, cells were harvested, washed, stained with viability dye and antibodies against the flow cytometry panel included Live/Dead‐BV510, CD45.2‐BUV395, TCRβ‐BUV737, CD4‐BV605, CD8‐AF700, CD25‐APC‐Cy7, CD44‐FITC, CD62L‐BV650, PD‐1‐PE, and TNF‐α‐BV785. Tumor‐cell killing (48 h) was quantified by the relative reduction in viable CellTrace Violet‐positive AT3‐OVA tumor cells, together with assessment of OT‐I T‐cell activation and effector phenotypes.

### 5.22 Bone Marrow‐Derived Macrophage Culture and Stimulation With AT3‐OVA Conditioned Medium

Bone marrow was flushed from the long bones of adult C57BL/6J mice using phosphate‐buffered saline (PBS) containing 2% fetal bovine serum (FBS). To generate bone marrow‐derived macrophages (BMDMs), approximately 1 × 10^7^ bone marrow cells were seeded on bacteriological plastic dishes in complete RPMI medium supplemented with 10% FBS, 25 U/mL penicillin, 25 µg/mL streptomycin (Gibco, Thermo Fisher Scientific, Australia), and recombinant CSF1 (100 ng/mL). Cells were differentiated for 7 days, mechanically detached, and reseeded into 6‐well plates at 2.5 × 10^6^ cells per well.

Conditioned medium was generated from AT3‐OVA derivatives (C7, C8 clones) by culturing 1 × 10^7^ tumor cells per condition for 3 days. Culture supernatants were collected, clarified to remove cellular debris, and concentrated 1:10 prior to use for macrophage stimulation. Once BMDMs reached confluence, culture medium was replaced with 2.5 mL stimulation medium consisting of 1.25 mL RPMI containing 25 U/mL penicillin and 25 µg/mL streptomycin, and 1.25 mL concentrated AT3‐OVA‐ derivatives derived conditioned medium. BMDMs were treated for 72 h before downstream gene expression analysis.

### 5.23 Gene Expression Analysis

Following conditioned‐medium treatment for 72 h, BMDMs were collected in TRIzol reagent (Sigma‐Aldrich), and RNA extraction and cDNA synthesis were performed according to the manufacturers’ instructions using Bioline reagents. Quantitative PCR was performed using SYBR Select Master Mix (Thermo Fisher Scientific) on an Applied Biosystems QuantStudio system. Relative gene expression was assessed using the following primer pairs: Hprt forward, 5′‐GCAGTACAGCCCCAAAATGG‐3′ and reverse, 5′‐AACAAAGTCTGGCCTGTATCCAA‐3′; Retnla forward, 5′‐AGGAACTTCTTGCCAATCCAGCT‐3′ and reverse, 5′‐GCAGGAGGCCCATCTGTTCA‐3′; Mrc1 forward, 5′‐CACACTCATCCATTACAACCAAAGCTG‐3′ and reverse, 5′‐GACCACGGTGACCACTCCTG‐3′; Il1b forward, 5′‐CAACCAACAAGTGATATTCTCCATG‐3′ and reverse, 5′‐GATCCACACTCTCCAGCTGCA‐3′; and Cxcl10 forward, 5′‐GCCCACGTGTTGAGATCATTGC‐3′ and reverse, 5′‐GTGCGTGGCTTCACTCCAGT‐3′.

IL‐10 F AGCTCCAAGACCAAGGTGTC, IL‐10 R TCCAAGGAGTTGTTTCCGTTA

Tnf_F: GGTCCCCAAAGGGATGAGAAG, Tnf_R: TCGAATTTTGAGAAGATGATCTGAGTG

### 5.24 Statistical Analysis

Analyses included Student's *t*‐test, ANOVA, Bonferroni post‐tests, and log‐rank tests using GraphPad Prism (RRID:SCR_002798) v7.0 software. Significance was defined as: ns (*p* > 0.05), * (*p* ≤ 0.05), (*p* ≤ 0.01), * (*p* ≤ 0.001), and **** (*p* ≤ 0.0001). Results were expressed as mean ± SD.

## Author Contributions

Conceptualization: B.R., P.M.A., K.K.; Investigation: B.R., P.T., P.A., B.B., B.B.W., S.I., S.K., N.J.W.H., P.M.A.; Bioinformatics: B.R., Y.Y., C.V., S.M.T., B.B.W., Z.X., H.V., P.H.G.D., P.M.A., Q.N.; Supervisory team: A.L.B., J.A.L., B.R., P.M.A., K.K.; Histology and Pathology: S.S., Z.X., A.S., J.F.; Data analysis: B.R., B.B.W., A.P., P.M.A.; Writing the original draft: B.R., A.L.B., P.M.A., K.K.; Review and Editing: B.R., S.T., P.M.A., K.K.; All authors read and approved the final manuscript.

## Funding

This work was supported by National Health & Medical Research Council (NH&MRC) Program Grant [ID 1017028] to KK. KK's lab is also supported by US DoD CDMRP Grant. BR was supported by GUPRS and GUIPRS from Griffith University and an Investigator Initiated Research Scheme (2023/IIRS0077) from the National Breast Cancer Foundation (NBCF), Australia. AP was supported by Victorian Cancer Agency Mid‐Career Research Fellowships (MCRF20027).

## Patient/Participant Consent Statement and Clinical Trial Registration

This study did not involve human participants / identifiable patient data; therefore, informed consent was not required. Also, we did not involve a clinical trial, prospective enrolment of human participants, or assignment of participants to a health‐related intervention. Therefore, a clinical trial registration number was not required.

## Conflicts of Interest

The authors disclose no potential conflicts of interest

“Rashidieh, Ben; Ziegman, Rebekah; Khanna, Kum Kum (2025). Genetic Ablation and Multi‐Omics Profiling Reveal CEP55 as a Key Driver of Tumorigenesis in Diverse Cancer Models.”

DOI: 10.17632/kkmmx9xnp4.1

DOI: 10.17632/ykffbkx4bs.1

DOI: 10.17632/49ggr7f2dc.1

Additional data and analysis code supporting the findings of this study are available from the corresponding authors upon reasonable request.

## Supporting information




**Supporting File 1**: advs76782‐sup‐0001‐SuppMat.docx.

**Supporting File 2**: advs76782‐sup‐0002‐TableS1‐S6.docx.

**Supporting File 3**: advs76782‐sup‐0003‐Data.zip.

## Data Availability

The data that support the findings of this study are available within the article and its Supporting Information. The proteomic and phosphoproteomic datasets generated in this study are openly available in Mendeley Data at:
