# Supplementary material for: Genetic Ablation and Multi‐Omics Profiling Reveal CEP55 as a Key Driver of Tumorigenesis in Diverse Cancer Models
Source: Adv Sci (Weinh). 2026 Aug 10;13(58):e76782. doi: 10.1002/advs.76782 (PMC13455837; doi:10.1002/advs.76782)
Supplement: Supplementary file 1 [file ADVS-13-e76782-s001.docx]

Supplementary Tables

| **Cluster (Significantly Correlated with CEP55 Expression in cancers-TCGA)** | **Pathways (TCGA across top 20 cancers)** |
| --- | --- |
| **Cell Cycle** | P53 Signaling RB regulation E2F TF network FOXM1 network Cell cycle /checkpoints events Cytokinesis and mitotic events Telomerase events Sonic Hedgehog regulating cell cycle |
| **DNA Damage** | DNA damage signaling DNA replication initiation ATM/ATR signaling MRN complex relocation RAD50/51, MRE11, NSB1 complex events DNA double strand break events BRCA events |
| **Signaling** | Myc pathway ( TF mediated and signaling) P38 MAPK events PLK events Aurora signaling Insulin signaling |
| **Immunity** | IL-6 signaling IL-2 Signaling mediated by PI3K MHC II antigen presentation TNF stress related signaling |
| **Apoptosis** | Caspase Cascade Apoptotic events Proteasome complex events Ubiquitin degradation CDC25A/Cyclin D1 events |

**ST1. TCGA pathways correlated with CEP55 Expression**

**ST1**

| **Cluster (UPREG SV40-MEFs KO/Wt )** | **Pathways (UPREG SV40-MEFs KO/Wt)** |
| --- | --- |
| **Cellular response** | Nutrition Starvation Corticosteroids Oxidative stress Hormones |
| **Differentiation** | Tissue regeneration Skin Nitric oxide regulation T-cell differentiation |
| **Tissue growth** | Fibroblast growth  Stellate activation Multicellular organism growth Collagen biosynthesis Integrin-mediated adhesion |
| **Actin**   **filament**   **organization** | Actin cytoskeleton regulation  Endoderm formation Extracellular matrix organization Selective autophagy |
| **Signal transduction** | Response to transforming growth factor beta (TGFβ)  Tumor necrosis factor (TNF) |
| **Metabolism** | Cholesterol Hexose Amino acids |

**ST2. Differentially Upregulated pathways in Cep55 SV40-MEFS**

**ST2**

**ST3. Differentially Up & downregulated phosphosites in E1A-RAS Cep55 KO MEFS**

**ST3**

| **Query A** | **Query B** | **Log2**  **Odds Ratio** | **p-Value** | **q-Value** | **Tendency** |
| --- | --- | --- | --- | --- | --- |
| ITGB1: AMP GAIN | CEP55: AMP GAIN | >3 | <0.001 | <0.001 | Co-occurrence |
| PTK2: AMP GAIN | CEP55: EXP >1.5 | 1.804 | 0.004 | 0.024 | Co-occurrence |
| ITGAV:H0MDEL HETLOSS | CEP55: EXP >1.5 | 2.057 | <0.001 | 0.007 | Co-occurrence |
| ITGA5:H0MDEL HETLOSS | CEP55: H0MDEL HETLOSS | 2.271 | <0.001 | <0.001 | Co-occurrence |
| ITGB1: HOMDEL HETLOSS | CEP55: H0MDEL HETLOSS | >3 | <0.001 | <0.001 | Co-occurrence |
| ITGAV= integrin αv ITGA5= integrin α5 ITGB1= integrin β1 FN1: Fibronectin PTK2: Focal adhesion kinase (FAK) AMP/GAIN: Increased Copy number EXP>1.5 mRNA expression is 1.5 SD above the mean EXP<1.5 mRNA expression is 1.5 SD below the mean | | | | | |

**ST4. Correlative Analysis of Indicated Gene Alterations & CEP55 Dysregulation**

**ST4**

Pten KD

Cep55-Pten DKD

| **Genotype** | **Cep55-Pten DKD** | **Pten KD** |
| --- | --- | --- |
| **Lymphoma** | 11(16) | 6(8) |
| **Extra-medullary hemopoiesis** | 4(16) | 1(8) |
| **Squamous papilloma** | 4(16) | 0 |
| **Hematoma** | 5(16) | 1(8) |
| **Hemangio-endothelioma (sarcoma)** | 2(16) | 0 |

**ST5. The observed pathological defects in Cep55-Pten DKD and Pten KD mice**

**ST5**

|  | Cep55-Pten | Pten |
| --- | --- | --- |
| Lymphoma | 68.75 | 88.88 |
| Extramedullary Hemopoiesis | 25 | 5.55 |
| Squamous Papilloma | 25 |  |
| Hematoma | 31.25 | 5.55 |
| Sarcoma | 12.5 |  |

|  | Pten |
| --- | --- |
| Lymphoma | 88.88 |
| Extramedullary Hemopoiesis | 5.55 |
| Hematoma | 5.55 |

| **DOWNregulated Clusters:** | **Biological Processes** |
| --- | --- |
| **Adhesion** | Epithelial and endothelial migration Adhesion spreading substrate  Integrin-mediated signaling pathway  Integrin substrate spreading |
| **Actin Filament polymerization or stabilization** | actin filament polymerization, negative regulation of depolymerization |
| **Endocytic events** | Regulation of endocytosis,recycling, phagocytosis, internalization, autophagy, lysosomes  ERK1/ERK2 cascade  Endosomal transport receptor signaling pathway |
| **Angiogenesis** | Positive regulation of angiogenesis |
| **Leukocytes** | Chemotaxis leukocytes Leukocyte activation involved in immune response Regulation of leukocyte migration |
| **UPregulated Clusters:** | **Biological Processes** |
| **Purine biosynthesis** | Purine nucleoside tri/di-phosphate metabolic process ATP generation from ADP Electron transport chain |
| **transition cycle phase,** **sister chromosome segregation** | Regulation of mitotic cell cycle G2 transition cycle Mitotic G1 DNA damage checkpoint signaling Spindle assembly Regulation of chromosome organization |
| **mRNA translation** | Negative regulation of translation, mRNA stability, Regulation of mRNA catabolic process |

**ST6. E1A/Ras KO MEFs & Cep55-Pten DKD**

**ST6**
