# Supplementary material for: Genetic Ablation and Multi‐Omics Profiling Reveal CEP55 as a Key Driver of Tumorigenesis in Diverse Cancer Models
Source: Adv Sci (Weinh). 2026 Aug 10;13(58):e76782. doi: 10.1002/advs.76782 (PMC13455837; doi:10.1002/advs.76782)
Supplement: Supplementary file 2 [file ADVS-13-e76782-s003.docx]

**Genetic Ablation and Multi-Omics Profiling Reveal CEP55 as a Key Driver of Tumorigenesis in Diverse Cancer Models.**

Behnam Rashidieh^1,2*^, Yuanhao Yang^1^, Amanda Louise Bain^2^, Phillip Theron^1,2^, Catharina Vosloo^1^, Sahar Keshvari^1^, Parinaz Ahangar^3^, Brydie Bowden^4^, Simon M. Tria^2^, Ben B. Wang^5,6^, Sandra Isenmann^6,7^, Nicholas J. Westra van Holthe^3,8^, Zherui Xiong^2^, Hani Vu^2^, Sowmya Sharma^2^, Asmerom Sengal^1^, J. Alejandro Lopez^2,4^, John Finnie9, Antonella Papa ^10,11^ , Pascal HG Duijf^12^, Quan Nguyen^2^, Pirjo M Apaja^5,6,7^*, Kum Kum Khanna^1,2^*

^1^ Mater Research Institute, The University of Queensland, Translational Research Institute, Brisbane, QLD 4102, Australia.

^2^ QIMR Berghofer Medical Research Institute, Brisbane, QLD 4006, Australia

^3^ Australian Institute for Bioengineering and Nanotechnology, The University of Queensland, Brisbane, Queensland 4072, Australia.

^4^ Griffith University, Brisbane, QLD 4111, Australia

^5^ University of Adelaide, Department of Molecular & Cellular Biology, School of Biological Sciences, Adelaide, SA, 5000, Australia.

^6^ South Australian Health and Medical Research Institute, Adelaide, SA, 5000, Australia.

^7^ Flinders University, College of Public Health and Medicine, Bedford Park, SA, 5042, Australia.

^8^ ARC Centre of Excellence in Quantum Biotechnology, St Lucia, Queensland, Australia

^9^School of Biomedicine, Faculty of Health and Medical  Sciences, University of Adelaide, Adelaide SA

^10^The University of Adelaide, South Australian immunoGENomics Cancer Institute, Adelaide, South Australia, Australia.

^11^ Monash Biomedicine Discovery Institute and Department of Biochemistry and Molecular Biology, Monash University, Melbourne, VIC, 3800, Australia.

^12^ Centre for Cancer Biology, Clinical and Health Sciences, University of South Australia & SA Pathology, Adelaide, SA 5001, Australia

**Supplementary Figures**

**Supplementary figure 1. CEP55 is highly expressed across multiple human cancers and correlates with poor patient survival, multiple oncogenic pathway activation, and immune dysregulation.**

(A) CEP55 RNA expression across selected TCGA tumors vs matched normal tissues. (B) Kaplan–Meier curves showing overall and disease-free survival in patients stratified by high vs low CEP55 expression (see associations between CEP55 and clinical features in Sup material_1). (C, D) Correlation of CEP55 expression with top 5 oncogenic signatures (based on 1387 cancer-related features; see Sup material_2), immune infiltration profiles, and immune checkpoint gene expression (CIBERSORT: see Sup material_3) across top 5 cancers. (E) CEP55 expression correlation with immune cell alterations (TISIDIB: see Sup material-1).

**Supplementary figure 2. Proteomic profiling of primary (p-MEFs), SV40-MEFs and E1A/Ras MEFs upon Cep55 loss.**
(A) Venn diagram showing overlap of identified total and phosphoproteins. (B) Volcano plot of differentially expressed proteins in p-MEFs comparing Cep55 KO vs. Wt. (C, D) Correlation plots and normalization of proteomic intensities across samples. (E-G) Gene ontology (GO) enrichment analysis of significantly regulated proteins (using Fisher’s exact test) in Cep55 KO/Wt primary p-MEFs (e), SV40-MEFs (F), and E1A/Ras-MEFs (g). Data highlight stage-specific rewiring of ECM remodeling, integrin signaling, and ribosome biogenesis upon Cep55 loss.

**Supplementary figure 3. Distinct total and phosphoproteomic changes upon Cep55 loss.**
(A-D) GO pathway enrichment analysis of differentially expressed proteins in Cep55 knockout (KO) and wild-type (Wt) MEFs highlighting enrichment of (A, B) metabolic pathways and (C, D) molecular components; (E–H) Comparative expression analysis of integrin receptor subunits (E, F) and collagen chain genes (G, H).

**Supplementary Figure 4. Cep55 loss alters endosomal and lysosomal trafficking networks during oncogenic progression in MEFS.**

(A–C) Heatmaps of differentially expressed proteins (log₂ KO/Wt) involved in membrane transport and vesicular trafficking across a) primary (P-MEFs), (B) SV40-transformed, and (C) E1A/Ras-transformed MEFs. Categories include ESCRT complexes, endocytosis, retromer, lysosomal enzymes/receptors/transporters, cholesterol metabolism, and integrin trafficking. Upregulated proteins are shown in red and downregulated proteins in blue. Notable changes include ESCRT-I/III components (e.g., CHMP4B, VPS4A), lysosomal enzymes (e.g., LAMP1/2, GBA), and integrin trafficking regulators.

**Supplementary Figure 5. Integrated proteomic and phosphoproteomic analyses reveal Cep55-dependent alterations in integrin signaling, Focal adhesion, cytoskeletal remodeling, and chromatin regulation.**
*(A, B)* Gene Ontology (GO) enrichment of significantly altered total proteins in *E1A/*Ras MEFs (Cep55 KO vs Wt) highlighting changes in metabolic (A) and molecular component (B). Downregulated pathways include platelet activation, endocytosis and focal adhesion, while upregulated terms involve oxidative phosphorylation and chromatin organization. *(C, D)* Changes in Integrin-associated receptors and tetraspanin networks in *E1A/*Ras MEFs, showing reduced expression of laminin and RGD-binding integrins (*α3β1*, *α5β1*, *α6β4*) and decreased *Cd63* and *Tspan6* levels, consistent with impaired integrin trafficking and matrix interaction. *(E)* Changes in collagen chains in *E1A/*Ras MEFs. *(F,G)* GO enrichment of pathways and molecular components significantly altered phosphoproteins, showing downregulation of pathways associated with DNA-templated transcription, RNA processing, chromatin remodeling, and VEGFR2 permeability while upregulating cell-cycle and SUMOylation-related pathways in Cep55 KO vs Wt *E1A/*Ras MEFs.
*(H)* Heatmap of top differentially phosphorylated proteins in Cep55 KO vs Wt *E1A/*Ras MEFs, including *Frs2*, *Bin1*, *Bcr*, and *Dnmt1*, representing key regulators of receptor signaling and epigenetic control. *(I)* Comparative phosphosite expression profiles across *p-MEFs*, *SV40 MEFs*, and *E1A/*Ras *MEFs* highlighting progressive activation of phosphorylation events during oncogenic transformation, notably at *Tnks1bp1*, *Hmgp1*, *Myh9*, and *Srrm1*.

**Supplementary Figure 6. Loss of Cep55 disrupts integrin expression and endocytosis, downstream signaling, and cell motility.**
*(A)* Immunofluorescence images showing α5-integrin (green) and early endosome antigen 1 (EEA1, magenta) co-localization in SV40 MEFs 15 minutes post-internalization. Cep55 knockout (KO) cells display higher α5-integrin internalization compared with wild-type (WT) controls. *(B)* Flow cytometry analysis (histogram overlays) confirming higher surface expression of α- and β-integrin subunits in Cep55 *KO* SV40 MEFs compared with Wt lines. *(C, D)* Immunoblot analyses of SV40 (c) and E1A/Ras (d) MEFs showing reduced activation of signaling molecules such as phosphorylation of AKT (Ser473), ERK1/2 (Thr202 and Tyr204), FAK (Tyr397), and Paxillin (Tyr118), along with reduced total level of RHOA and PLA2G4 in E1A/Ras *Cep55* KO cells compared to Wt. These findings indicate impaired integrin-mediated signaling and cytoskeletal organization. *(E)* Time-lapse single-cell tracking (Holomonitor) showing attenuated motility but not motility speed in *Cep55*⁻/⁻ SV40 MEFs relative to Wt. Quantified data represent mean ± SD. Holomonitor live-cell tracking analysis was performed on >20 individual cells per condition. Representative cell tracks (n = 5) are shown. (***P < 0.001; ns=not significant*).
*(F)* TCGA breast cancer dataset analysis demonstrating co-occurrence (see Sup T4) between *CEP55* amplification and key integrin signaling components (*ITGAV*, *ITGB1, ITGA5*, *FAK(PTK2)*, *Fibronectin*), along with HER2-positive and ER/PR-indeterminate clinical subgroups, supporting CEP55’s clinical association with integrin-driven oncogenic signaling.

**Supplementary Figure 7. Generation and validation of the conditional *Cep55* knockout (cKO) mouse model demonstrating its dispensability for adult homeostasis.**
*(a)* Schematic of the Cep55 gene targeting strategy showing the wild-type (*Cep55*⁺/⁺), gene-trapped knockout-first (tm1a) allele containing *LacZ* and *neo* cassettes, and the conditional floxed (tm1c) allele following *Flp*-mediated recombination. *LoxP* and *FRT* sites are indicated, and primer locations for genotyping are shown by blue arrows ( P1,2,3 and Rec). *(b)* Breeding and genotyping workflow for generating tamoxifen-inducible *Cep55* conditional knockout (*Cep55*^Fl/Fl; Cre^ERT2⁺) mice. Strategy includes sequential crosses to remove *Flp* and obtain the floxed allele, followed by *Rosa26-CreERT2* integration and confirmation of recombination efficiency by PCR with expected band sizes. *(c)* *Cep55-*RNA expression across mouse tissues (embryonic, juvenile, and adult stages), showing the highest expression in testis (blue), with detectable levels in spleen (purple) and intestine (light blue), and low expression in lung (orange) in adult mice. *(d)* Immunoblot analysis of *Cep55* protein expression in adult wild-type tissues aligns with RNA data, showing elevated expression in testis, moderate levels in spleen and intestine, and low levels in lung (c). β-Actin serves as a loading control.
*(e)* Immunoblot validation of *Cep55* deletion following tamoxifen induction, demonstrating efficient loss of Cep55 in *Cep55* cKO compared to heterozygous (Het) and wild-type (Wt) controls. *(f)* Tamoxifen-induced deletion of *Cep55* in adult *Cep55*^Fl/Fl; Cre^ERT2⁺ mice causes no overt phenotype or gross morphological defects, with 100% survival over a 26-week observation period.

**Supplementary Figure 8. Cep55 loss modulates PI3K/AKT, ERK and β catenin in Pten-deficient tumor-prone mice.**
*(a)* Immunoblot analysis of Cep55 and Pten expression in liver, testis, and spleen tissues from tamoxifen-induced *Pten* KD and *Cep55/Pten* double KD (DKD) tumor-bearing mice and vehicle-treated control samples. Vinculin was used as a loading control. *(b)* Quantification of immunohistochemistry (IHC) H-scores for phosphorylated AKT (pAKT), phosphorylated ERK (pERK), and β-catenin in tumors. H-scores were determined by independent, blind investigations by pathologists. Right panel: scatter plot shows reduced pAKT, pERK, and β-catenin scores in the *Cep55/Pten* DKD compared to Pten tumors. The number of samples scored for each group is indicated in the figure. *(c)* Representative IHC images of liver and spleen sections showing pAKT, pERK, and β-catenin expression in *Pten* KD versus *Cep55/Pten* DKD mice in indicated organs. Cep55 loss reduces AKT and ERK activation and β-catenin nuclear localization. Scale bars = 100 μm. *(d)* IHC staining for focal adhesion kinase (FAK) in spleen sections shows attenuated FAK expression upon Cep55 loss in the *Cep55/Pten* DKD cohort. Insets show magnified areas. Scale bars = 100 μm.

**Supplementary Figure 9. Spatially resolved transcriptomic profiling reveals *Cep55*-dependent remodeling of tumor microenvironments in *Pten*-deficient tumors.**

(A) Violin plots showing spatial transcriptomic quality control metrics across tumor regions in Pten KD and Cep55/Pten DKD samples, including total transcript counts (nCount), detected gene features (nFeature), and hemoglobin (Hb%) content. Comparable sequencing depth and feature distribution confirm dataset uniformity and sample integrity. (b, c) Spatial mapping of Cep55 mRNA (B) and Cep55 protein (C) by spatial transcriptomics and IHC, showing marked downregulation in Cep55/Pten DKD tumors compared to Pten KD. (d, e) Spatial transcriptomics and IHC validation of Pten expression showing successful recombination and expected Pten loss in both Pten KD and DKD tumors. High-magnification insets highlight focal Pten-positive regions in *Pten* KD but are largely absent in DKD samples. (f) Co-mapping of Ptprc (CD45 isoform B220) with pathologist-annotated tumor regions and B220 (B-cell marker) expression by spatial transcriptomics and IHC. Results show altered lymphoid architecture and immune cell localization in DKD tumors, indicating Cep55-dependent modulation of the tumor immune landscape.

**Supplementary Figure 10. Comparative transcriptomic and pathway analyses reveal conserved Cep55-dependent signaling alterations between tumorigenic MEFs and Pten-deficient cancerous tissues.**

(a–c) Gene Ontology (GO) enrichment analyses of differentially expressed genes between Pten KD and Cep55/Pten DKD tumors reveal altered: (a) molecular functions, (b) metabolic pathways, and (c) unique biological processes including mitotic control, DNA repair, immune activation, and metabolic reprogramming (e.g., p38/MAPK, TORC1, myeloid and lymphocyte differentiation, upon Cep55 loss; (d) Volcano plots showing representative differentially expressed genes uniquely enriched in Pten KD (e.g., Dep1, Tspo, Ly6B) and Cep55/Pten DKD (Ccl4, Mmp2, Mmp12), across tumor (upper panel) and tumor and stroma (lower panel) samples, illustrating immune-matrix remodeling upon Cep55 loss. (e) Comparative heatmaps of shared and distinct pathway enrichments across E1A/Ras MEFs and Pten-deficient tumor tissues (Wt Vs KD) across stroma and tumor (right panel) and tumor only (left panel). Both systems show conserved regulation of cell–substrate adhesion, integrin-mediated signaling, and protein sumoylation, and interferon signaling. Cep55 upregulation promotes focal adhesion and extracellular matrix disruption, highlighting cross-model conservation of Cep55-regulated oncogenic and structural networks.

**Supplementary Figure 11. Transcriptomic and experimental framework for Cep55 -dependent immune interrogation in Pten-deficient tumor models.**

(a) Boxplots showing mean log₁₀-transformed UMI counts, with 95% confidence intervals, for selected differentially expressed genes comparing Pten-KD tumors (blue) and Cep55/Pten double-knockdown tumors (CP-DKD, orange). Cep55 loss was associated with reduced expression of adhesion-related genes and increased expression of immune-related transcripts. (b) Clustered pathway-signature heatmap across endosomal/vesicle-trafficking, immune-modulation, and integrin/adhesion categories comparing E1A/Ras MEFs, Pten-TMA tumor, and Pten stroma+tumor samples. (c) Heatmap of immune-module scores in tumor and tumor+stroma compartments from Pten-KD and CP-DKD samples. Blue indicates lower marker scores, whereas red indicates higher marker scores. Values represent mean normalized enrichment scores for each module. (d) Effect-size analysis of immune-module scores showing directional bias toward Pten-KD or CP-DKD samples. Values near zero indicate mixed or equivalent enrichment between groups. Blue indicates a substantial effect-size shift toward CP-DKD, whereas red indicates a shift toward Pten-KD. (e) Normalized enrichment scores for immune modules. (f) Schematic of the AT3-OVA/OT-I co-culture system, in which SIINFEKL peptide is presented by MHC class I to antigen-specific OT-I CD8 T cells. (g) Schematic of the doxycycline-inducible shRNA expression cassette (Dharmacon) used for Cep55 targeting. The construct includes the 5′ long terminal repeat (5′ LTR), Psi packaging signal (Ψ), Rev response element (RRE), Tet-On response element 3G (TRE3G), turbo red fluorescent protein (tRFP), SMARTvector shRNA scaffold containing the targeting sequence, species-specific promoter (hEF1α or mEF1α), puromycin resistance gene (PuroR), self-cleaving 2A peptide, Tet-On 3G tetracycline-regulated transactivator, woodchuck hepatitis virus post-transcriptional regulatory element (WPRE), and 3′ self-inactivating long terminal repeat (SIN LTR).

**Figure S12. CEP55 loss reshapes macrophage polarization and modulates anti-tumor immune responses in AT3-OVA cells.**

(a) Top panel- Representative morphology of AT3-C7 and AT3-C8 cells without doxycycline (Cep55 Wt) and with doxycycline-induced Cep55 knockdown ((Cep55 KD). Lower panel: Relative fold changes in the gene expression of the indicated macrophage-associated genes, determined by RT–qPCR. (b) Immunoblot showing CEP55 expression in AT3-C7 and AT3-C8 cells ± doxycycline; Vinculin, loading control. (c) OT-I killing assay performed over 48 h showing the AT3 tumor-cell fraction, identified by CellTrace Violet (CTV) staining, frequency of live CD8⁺ T-cell and proportion of TNFα⁺ and CD25⁺ (IL-2Rα) T cell subpopulations across C7 and C8 AT3-OVA derivatives with or without doxycycline treatment. Data are presented as mean ± SD; n = 4 per genotype/condition. Statistical significance was determined by ANOVA test. (d) Flow-cytometric gating strategy used for OT-I phenotyping. (e) OT-I immunophenotyping following 72 h co-culture of OT-I splenocytes with C7 and C8 AT3-OVA derivatives without or with doxycycline-induced Cep55 knockdown, showing the frequency of live CD8⁺ T cells, activated CD44⁺CD62L⁻ T-cell subsets (TEM: T cells effector-memory), and PD-1 expression. (f) Representative images of human B-cell lymphoma lines (Raji, Ramos, Daudi, and BJAB), transduced with either scrambled control adenovirus (Ad-U6-RNAi) or human CEP55-targeting shRNA adenovirus (GFP-tagged; MOI =200) showing GFP signal. (g) Immunoblot showing CEP55 expression following adenoviral transduction across indicated cell lines; Vinculin, loading control. (h) immunoblot showing expression of CEP55, MHC I, and PTEN following CEP55 knockdown in BJAB cells using siRNA (20 nM); GAPDH, loading control. (i) BJAB cell viability following CEP55 knockdown using CEP55 siRNA #1 and #3 knockdown.
