# Supplementary material for: Genetic Ablation and Multi‐Omics Profiling Reveal CEP55 as a Key Driver of Tumorigenesis in Diverse Cancer Models
Source: Adv Sci (Weinh). 2026 Aug 10;13(58):e76782. doi: 10.1002/advs.76782 (PMC13455837; doi:10.1002/advs.76782)
Supplement: Supplementary file 3 [file ADVS-13-e76782-s002.zip › advs76782-sup-0002-Data/Sup Material_1 (TISIDB).pdf]

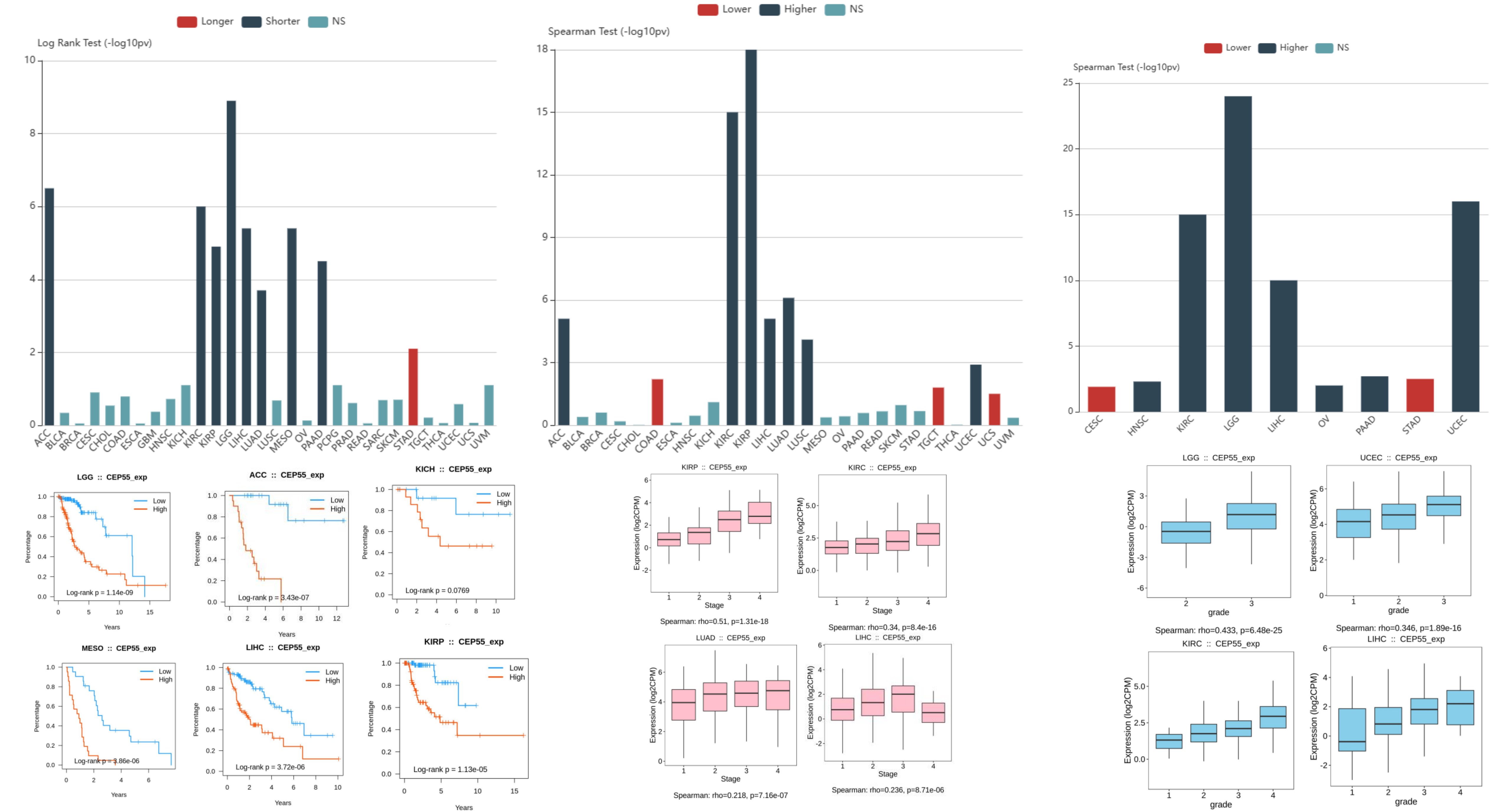

Fig a: Associations between CEP55 and clinical features: **Overall survival analysis** (left); **Cancer stage** (middle); **Tumor grade** (right)

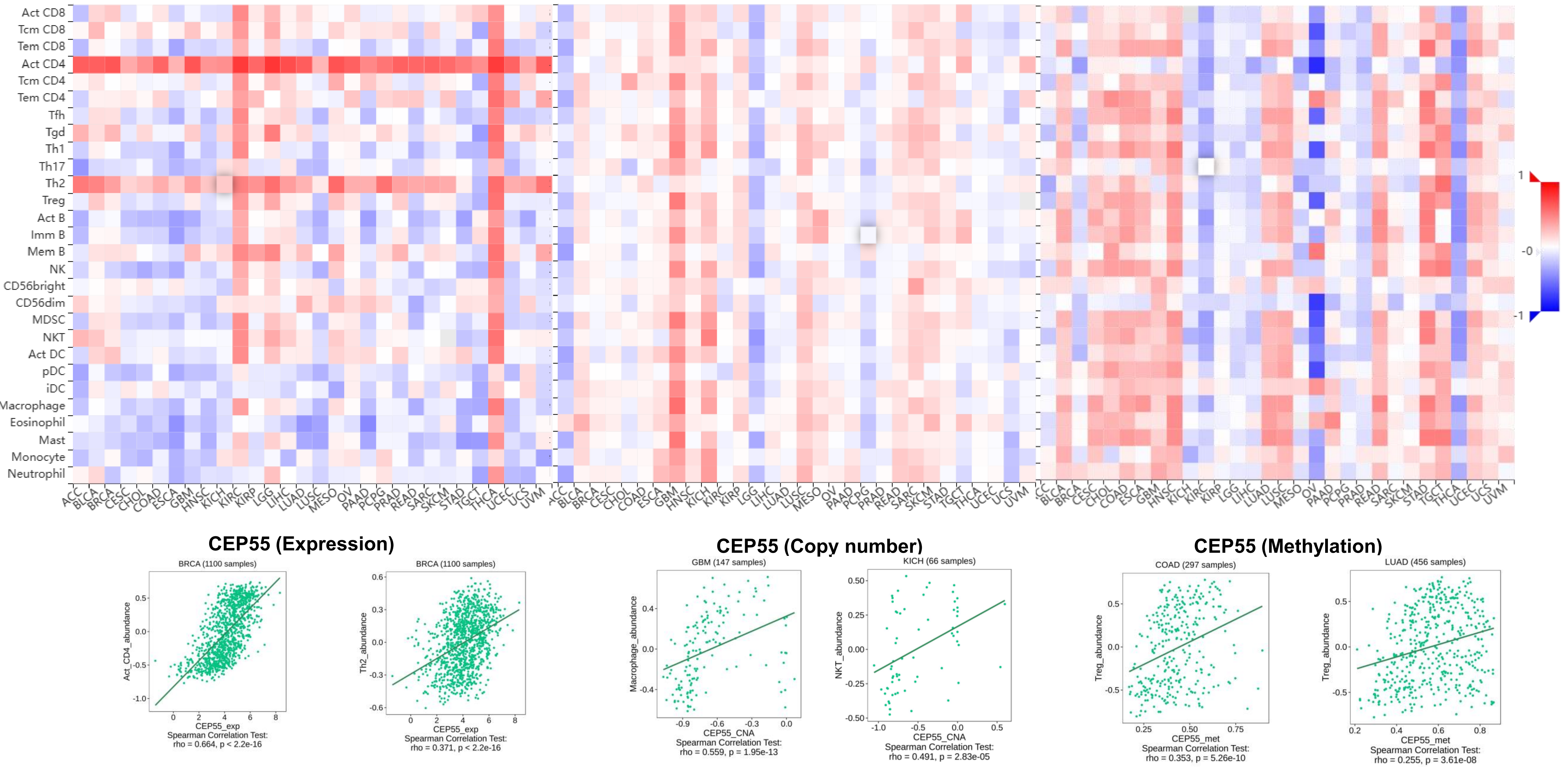

Fig b: Relations between abundance of tumor-infiltrating lymphocytes (TILs) and expression, copy number, methylation, of CEP55. The immune-related signatures of 28 TIL types from Charoentong's study, which can be viewed in the download page. For each cancer type, the relative abundance of TILs were inferred by using gene set variation analysis (GSVA) based on gene expression profile. In this tab, users can examine which kinds of TILs might be regulated by the current gene. (Source: **TISIDB**: an integrated repository portal for tumor-immune system interactions).

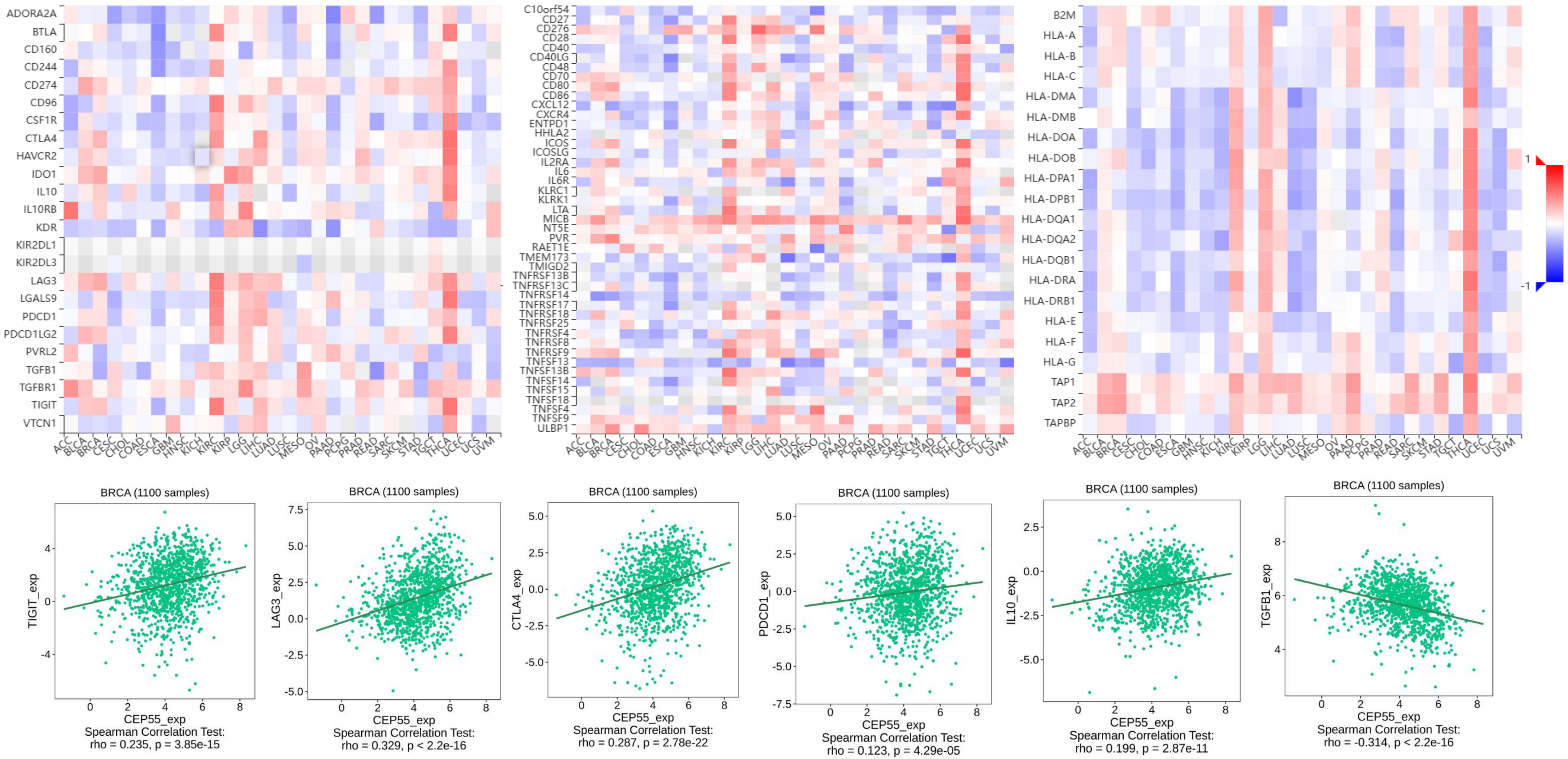

Fig c: Relations between three kinds of immunomodulators and expression of CEP55. These immunomo-dulators were collected from Charoentong's study. In this tab, users can examine which immunomodulators might be regulated by CEP55.: **Immunoinhibitor** (left); **Immunostimulator** (middle); **MHC molecule** (right). Positive correlation of TIGIT, LAG3 CTLA4,, PD1, and IL-10 and negative correlation of TGFβ1 with CEP55 expression.

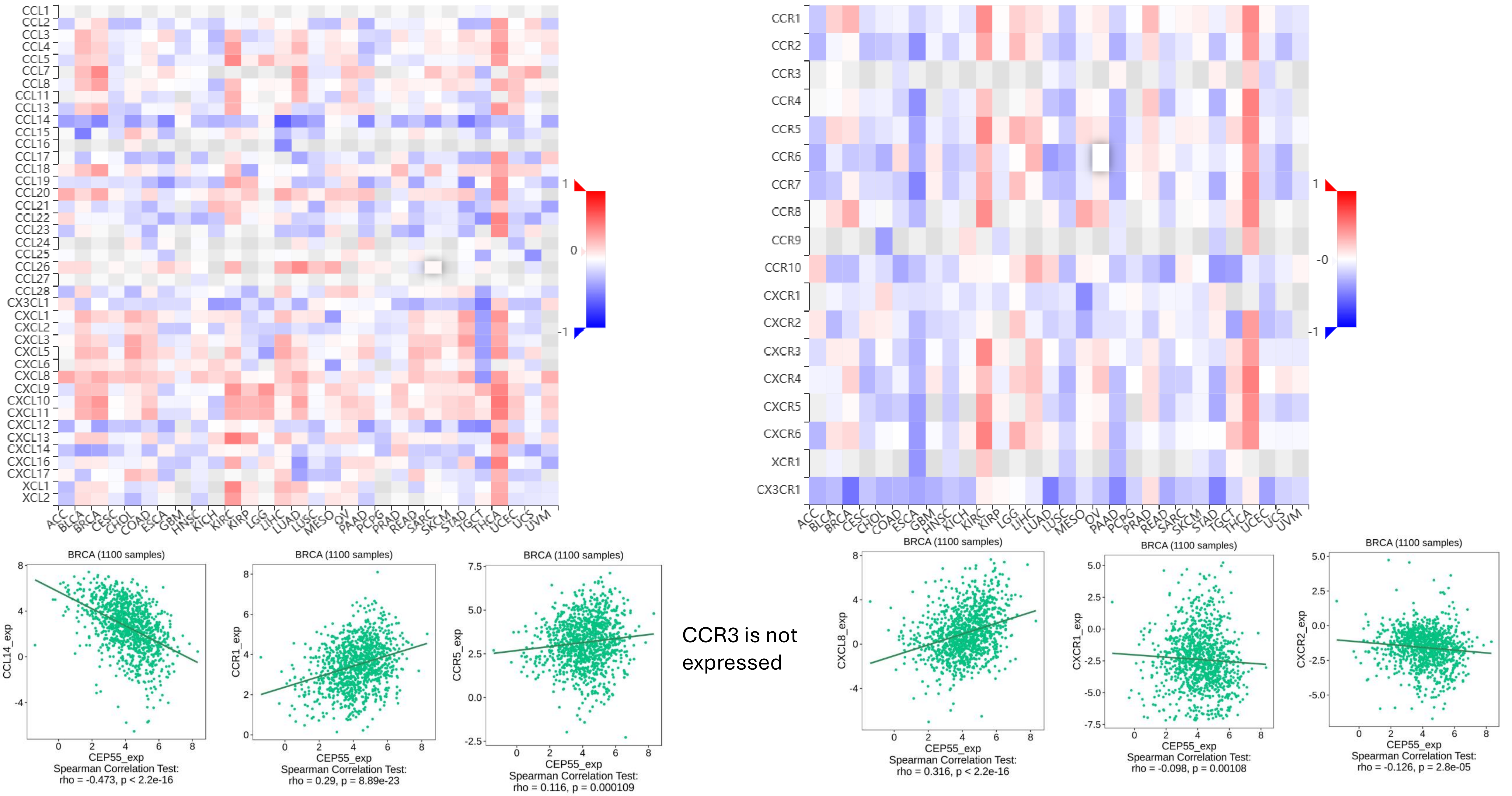

Fig d: Relations between chemokines (or receptors) and expression, of CEP55. In this tab, users can examine which chemokines (or receptors) might be regulated by the current gene: **Chemokine** (left); **Receptor** (right). Correlation study show the lowest and the highest chemokines (**CCL14**, AKA: HCC-1 and **CXCL8**, AKA: IL-8) and its related receptors correlation with CEP55 expression.

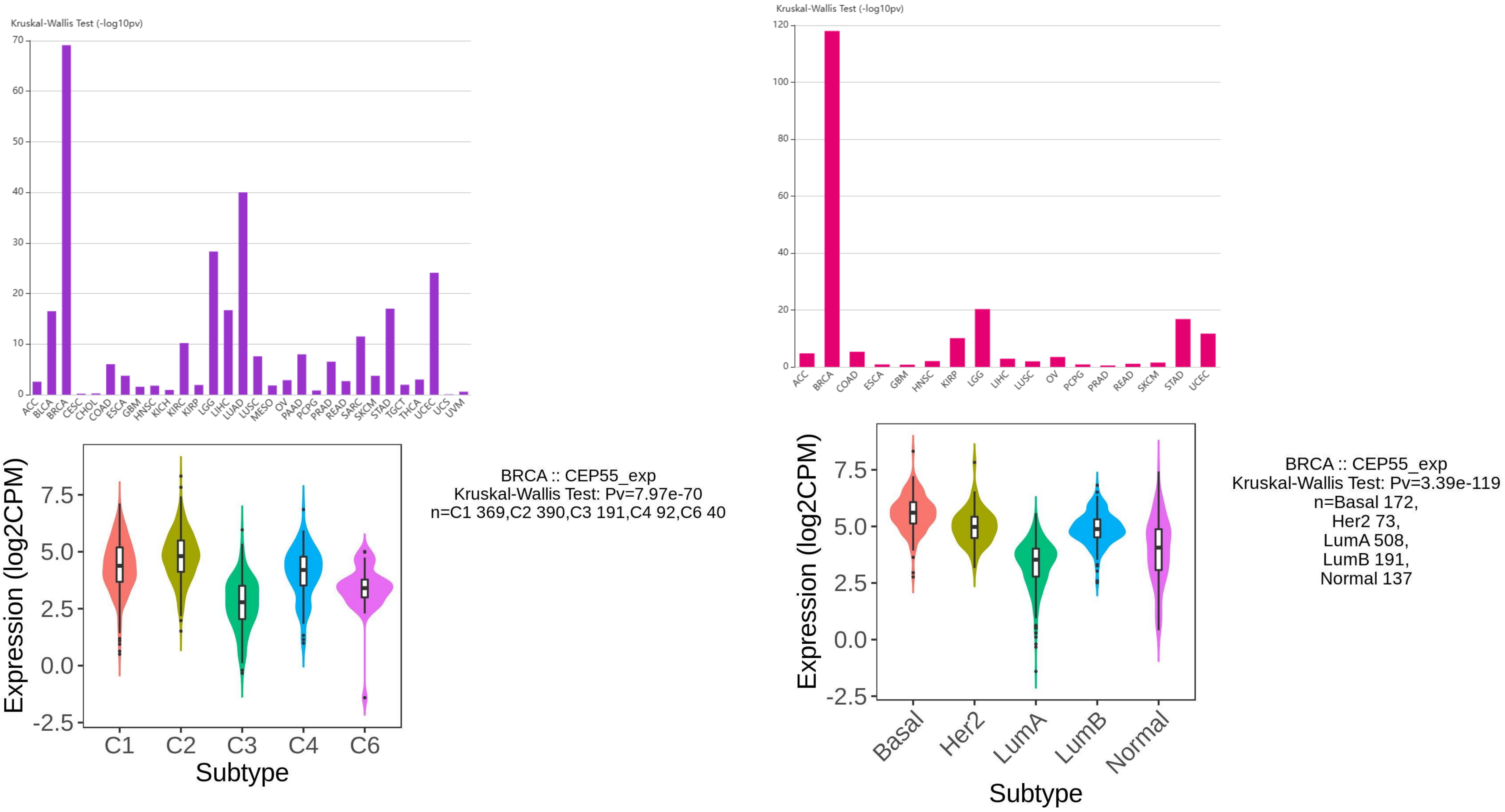

Fig e: Distribution of CEP55 expression across immune and molecular subtypes : **Immune subtype**: C1 (wound healing); C2 (IFN-gamma dominant); C3 (inflammatory); C4 (lymphocyte depleted); C5 (immunologically quiet); C6 (TGF-b dominant) and **Molecular subtype**
