# Supplementary material for: Genetic Ablation and Multi‐Omics Profiling Reveal CEP55 as a Key Driver of Tumorigenesis in Diverse Cancer Models
Source: Adv Sci (Weinh). 2026 Aug 10;13(58):e76782. doi: 10.1002/advs.76782 (PMC13455837; doi:10.1002/advs.76782)
Supplement: Supplementary file 3 [file ADVS-13-e76782-s002.zip › advs76782-sup-0002-Data/Sup Material_2 (1387_hallmarks_summary).pdf]

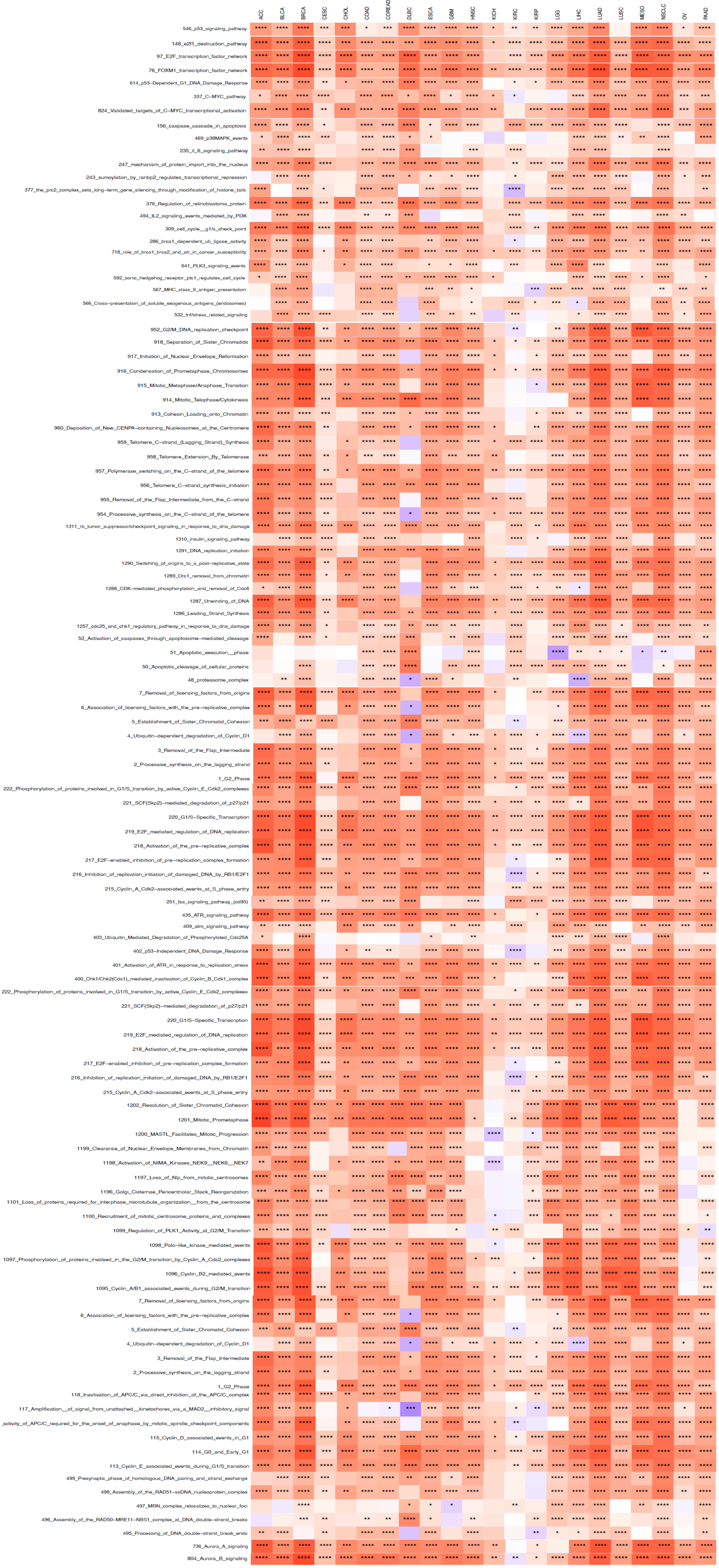
ACC
BLCA
BRCA
CESC
CHOL
COAD
COREAD
DLBC
ESCA
GBM
HNSC
KICH
KIRC
KIRP
LGG
LIHC
LUAD
LUSC
MESO
NSCLC
OV
PAAD
546_p53_signaling_pathway
148_e2f1_destruction_pathway
97_E2F_transcription_factor_network
76_FOXM1_transcription_factor_network
614_p53–Dependent_G1_DNA_Damage_Response
337_C–MYC_pathway
824_Validated_targets_of_C–MYC_transcriptional_activation
156_caspase_cascade_in_apoptosis
469_p38MAPK_events
235_il_6_signaling_pathway
247_mechanism_of_protein_import_into_the_nucleus
243_sumoylation_by_ranbp2_regulates_transcriptional_repression
377_the_prc2_complex_sets_long–term_gene_silencing_through_modification_of_histone_tails
376_Regulation_of_retinoblastoma_protein
494_IL2_signaling_events_mediated_by_PI3K
309_cell_cycle__g1/s_check_point
286_brca1_dependent_ub_ligase_activity
718_role_of_brca1_brca2_and_atr_in_cancer_susceptibility
641_PLK3_signaling_events
592_sonic_hedgehog_receptor_ptc1_regulates_cell_cycle
567_MHC_class_II_antigen_presentation
566_Cross–presentation_of_soluble_exogenous_antigens_(endosomes)
532_tnf/stress_related_signaling
952_G2/M_DNA_replication_checkpoint
918_Separation_of_Sister_Chromatids
917_Initiation_of_Nuclear_Envelope_Reformation
916_Condensation_of_Prometaphase_Chromosomes
915_Mitotic_Metaphase/Anaphase_Transition
914_Mitotic_Telophase/Cytokinesis
913_Cohesin_Loading_onto_Chromatin
960_Deposition_of_New_CENPA–containing_Nucleosomes_at_the_Centromere
959_Telomere_C–strand_(Lagging_Strand)_Synthesis
958_Telomere_Extension_By_Telomerase
957_Polymerase_switching_on_the_C–strand_of_the_telomere
956_Telomere_C–strand_synthesis_initiation
955_Removal_of_the_Flap_Intermediate_from_the_C–strand
954_Processive_synthesis_on_the_C–strand_of_the_telomere
1311_rb_tumor_suppressor/checkpoint_signaling_in_response_to_dna_damage
1310_insulin_signaling_pathway
1291_DNA_replication_initiation
1290_Switching_of_origins_to_a_post–replicative_state
1289_Orc1_removal_from_chromatin
1288_CDK–mediated_phosphorylation_and_removal_of_Cdc6
1287_Unwinding_of_DNA
1286_Leading_Strand_Synthesis
1257_cdc25_and_chk1_regulatory_pathway_in_response_to_dna_damage
52_Activation_of_caspases_through_apoptosome–mediated_cleavage
51_Apoptotic_execution__phase
50_Apoptotic_cleavage_of_cellular_proteins
48_proteasome_complex
7_Removal_of_licensing_factors_from_origins
6_Association_of_licensing_factors_with_the_pre–replicative_complex
5_Establishment_of_Sister_Chromatid_Cohesion
4_Ubiquitin–dependent_degradation_of_Cyclin_D1
3_Removal_of_the_Flap_Intermediate
2_Processive_synthesis_on_the_lagging_strand
1_G2_Phase
222_Phosphorylation_of_proteins_involved_in_G1/S_transition_by_active_Cyclin_E_Cdk2_complexes
221_SCF(Skp2)–mediated_degradation_of_p27/p21
220_G1/S–Specific_Transcription
219_E2F_mediated_regulation_of_DNA_replication
218_Activation_of_the_pre–replicative_complex
217_E2F–enabled_inhibition_of_pre–replication_complex_formation
216_Inhibition_of_replication_initiation_of_damaged_DNA_by_RB1/E2F1
215_Cyclin_A_Cdk2–associated_events_at_S_phase_entry
251_fas_signaling_pathway_(cd95)
435_ATR_signaling_pathway
409_atm_signaling_pathway
403_Ubiquitin_Mediated_Degradation_of_Phosphorylated_Cdc25A
402_p53–Independent_DNA_Damage_Response
401_Activation_of_ATR_in_response_to_replication_stress
400_Chk1/Chk2(Cds1)_mediated_inactivation_of_Cyclin_B_Cdk1_complex
222_Phosphorylation_of_proteins_involved_in_G1/S_transition_by_active_Cyclin_E_Cdk2_complexes
221_SCF(Skp2)–mediated_degradation_of_p27/p21
220_G1/S–Specific_Transcription
219_E2F_mediated_regulation_of_DNA_replication
218_Activation_of_the_pre–replicative_complex
217_E2F–enabled_inhibition_of_pre–replication_complex_formation
216_Inhibition_of_replication_initiation_of_damaged_DNA_by_RB1/E2F1
215_Cyclin_A_Cdk2–associated_events_at_S_phase_entry
1202_Resolution_of_Sister_Chromatid_Cohesion
1201_Mitotic_Prometaphase
1200_MASTL_Facilitates_Mitotic_Progression
1199_Clearance_of_Nuclear_Envelope_Membranes_from_Chromatin
1198_Activation_of_NIMA_Kinases_NEK9__NEK6__NEK7
1197_Loss_of_Nlp_from_mitotic_centrosomes
1196_Golgi_Cisternae_Pericentriolar_Stack_Reorganization
1101_Loss_of_proteins_required_for_interphase_microtubule_organization__from_the_centrosome
1100_Recruitment_of_mitotic_centrosome_proteins_and_complexes
1099_Regulation_of_PLK1_Activity_at_G2/M_Transition
1098_Polo–like_kinase_mediated_events
1097_Phosphorylation_of_proteins_involved_in_the_G2/M_transition_by_Cyclin_A_Cdc2_complexes
1096_Cyclin_B2_mediated_events
1095_Cyclin_A/B1_associated_events_during_G2/M_transition
7_Removal_of_licensing_factors_from_origins
6_Association_of_licensing_factors_with_the_pre–replicative_complex
5_Establishment_of_Sister_Chromatid_Cohesion
4_Ubiquitin–dependent_degradation_of_Cyclin_D1
3_Removal_of_the_Flap_Intermediate
2_Processive_synthesis_on_the_lagging_strand
1_G2_Phase
118_Inactivation_of_APC/C_via_direct_inhibition_of_the_APC/C_complex
117_Amplification__of_signal_from_unattached__kinetochores_via_a_MAD2__inhibitory_signal
_activity_of_APC/C_required_for_the_onset_of_anaphase_by_mitotic_spindle_checkpoint_components
115_Cyclin_D_associated_events_in_G1
114_G0_and_Early_G1
113_Cyclin_E_associated_events_during_G1/S_transition
499_Presynaptic_phase_of_homologous_DNA_pairing_and_strand_exchange
498_Assembly_of_the_RAD51–ssDNA_nucleoprotein_complex
497_MRN_complex_relocalizes_to_nuclear_foci
496_Assembly_of_the_RAD50–MRE11–NBS1_complex_at_DNA_double–strand_breaks
495_Processing_of_DNA_double–strand_break_ends
736_Aurora_A_signaling
804_Aurora_B_signaling
