# Supplementary material for: Genetic Ablation and Multi‐Omics Profiling Reveal CEP55 as a Key Driver of Tumorigenesis in Diverse Cancer Models
Source: Adv Sci (Weinh). 2026 Aug 10;13(58):e76782. doi: 10.1002/advs.76782 (PMC13455837; doi:10.1002/advs.76782)

# Immune cell infiltration (CEP55)

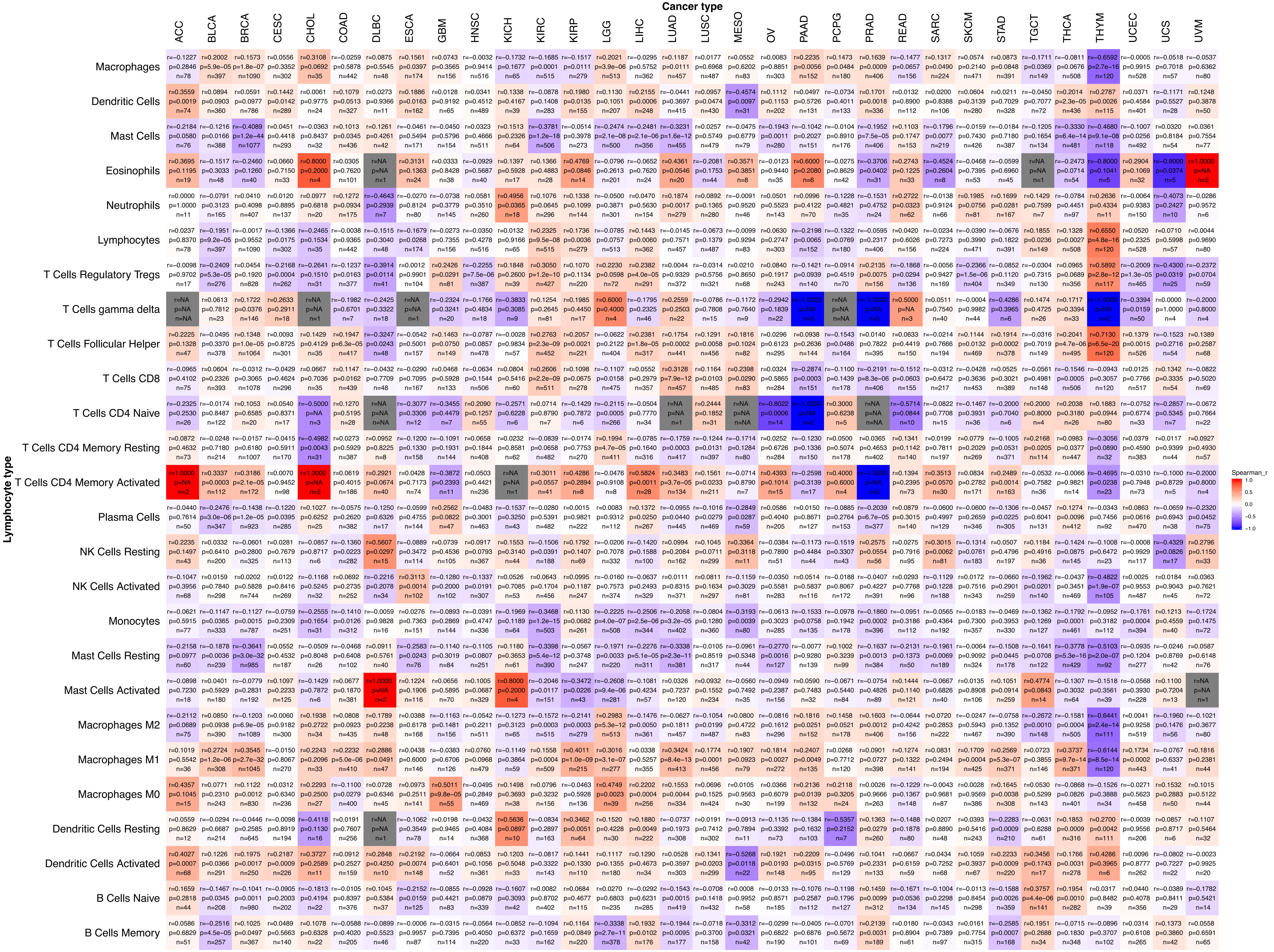

Supplement: Supplementary file 3 [file ADVS-13-e76782-s002.zip › advs76782-sup-0002-Data/Sup Material_3 (CIBERSORT).pdf]
